# Mechanistic insights into the T6SS of multi‐drug‐resistant *Aeromonas hydrophila* and its role in competition and pathogenesis

**DOI:** 10.1002/mlf2.70018

**Published:** 2025-07-22

**Authors:** Hao Wang, Ying Liu, Zhao Wang, PeiYi Xia, Zhiwei Li, Ming Liu, Yang Fu

**Affiliations:** ^1^ Department of Biochemistry School of Medicine, SUSTech Homeostatic Medicine Institute, Southern University of Science and Technology Shenzhen China; ^2^ The Third People's Hospital of Shenzhen the Second Affiliated Hospital of Southern University of Science and Technology Shenzhen China

**Keywords:** *Aeromonas* species, anti‐eukaryotic virulence, microbial interaction, T6SS

## Abstract

*Aeromonas hydrophila*, an opportunistic pathogen, often encodes Type VI Secretion System (T6SS) genes. However, the specific functions of T6SS, particularly in the context of clinical strains, remain poorly understood. In this study, we characterize a multi‐drug‐resistant strain, AH54, which possesses a complete and functional T6SS, composed of a structural cluster and two homologous auxiliary clusters (Aux1 and Aux2). Each auxiliary cluster encodes two distinct effector proteins: a rearrangement hotspot (Rhs) protein and a proline–alanine–arginine repeat (PAAR) protein—Rhs1/PAAR1 in Aux1 and Rhs2/PAAR2 in Aux2. Our findings reveal that AH54 assembles a fully operational T6SS capable of delivering these effectors, driving inter‐bacterial antagonism. Interestingly, the T6SS activity in AH54 is temperature‐regulated, with enhanced secretion and antibacterial activity at lower temperatures. To protect itself from self‐intoxication, AH54 produces immunity proteins (Tsi1–Tsi4) that neutralize the toxic effectors. While PAAR1 and PAAR2 are critical for Hcp secretion, immunity proteins Tsi3 and Tsi4 do not cross‐protect against PAAR effectors, suggesting distinct roles for each PAAR protein in optimizing AH54's competitive fitness. In addition, using a *Dictyostelium discoideum* phagocytosis model, we demonstrate that Rhs2, a metal ion‐dependent DNase effector, plays a crucial role in protecting AH54 from eukaryotic predation via T6SS. These findings highlight the pivotal role of T6SS in bacterial competition and pathogenesis, offering new insights into the virulence mechanisms of *A. hydrophila*.

## Impact statement

The Type VI Secretion System (T6SS), commonly used by Gram‐negative bacteria, drives interbacterial competition and contributes to eukaryotic virulence. In the context of multi‐drug‐resistant *Aeromonas hydrophila*, a significant clinical threat, the functional characterization of T6SS is essential for understanding its role in bacterial pathogenesis. In this study, we uncovered a unique genetic architecture of T6SS in the drug‐resistant strain AH54, revealing two homologous auxiliary clusters that encode four distinct effector proteins with potent activity against both prokaryotic and eukaryotic competitors. Notably, the Rhs2 effector shows dual‐target metal‐dependent DNase activity, providing a mechanism for resistance against eukaryotic predation. Our findings not only expand the understanding of T6SS diversity in *Aeromonas* species but also offer new insights into its contribution to bacterial competition and host–pathogen interactions, with implications for the development of targeted antimicrobial strategies.

## INTRODUCTION

*Aeromonas hydrophila* is a versatile aquatic pathogen capable of causing a wide range of infections in both humans and aquatic animals^1^. Transmission occurs primarily through contaminated food and water. In aquatic animals, it leads to motile *Aeromonas* septicemia (MAS), which causes significant economic losses in aquaculture^2^. In humans, *A. hydrophila* can result in gastroenteritis, septicemia, osteomyelitis, peritonitis, and tissue infections^3^, ^4^. This pathogen harbors numerous virulence factors, including aerolysins, hemolysins, adhesins, tissue‐destructive enzymes, S‐layers, iron‐binding systems, and protein secretion systems, such as Type III and Type VI secretion systems (T3SS and T6SS)^5^, ^6^. These secretion systems play a critical role in bacterial virulence, enabling interactions with both microbial competitors and host cells by delivering a wide array of effector proteins^7^.

T6SS, structurally similar to bacteriophage contractile injection systems, was first identified in *Vibrio cholerae* and *Pseudomonas aeruginosa* ^7^, ^8^ and is composed of three main complexes: a membrane complex, a baseplate, and a contractile sheath^9^. The sheath structure features an internal tube formed by stacked Hcp hexamers, topped by a spike of VgrG trimers, which are decorated with effector proteins and/or PAAR proteins^10^. Surrounding this tube is a contractile sheath made of polymerized VipA and VipB subunits^11^. Another essential component of T6SS is ClpV, an ATPase that provides the energy necessary for sheath contraction and effector injection^12^. T6SS effectors are critical to its function and are typically classified into two categories: specialized and cargo effectors. Specialized effectors possess a toxic C‐terminal domain covalently linked to structural proteins such as Hcp, VgrG, or PAAR, with their translocation coupled to the secretion of these components. In contrast, cargo effectors are independent toxic units whose secretion relies on interactions with structural proteins, often facilitated by chaperones^8^, ^13^, ^14^, ^15^. Recent studies have revealed diverse mechanisms by which T6SS effectors target and damage prey cells, including disruption of membranes, cell walls, nucleic acids, and metabolic proteins^16^, ^17^, ^18^. To prevent self‐intoxication, bacteria produce cognate immunity proteins that neutralize the toxicity of effectors; these immunity proteins are encoded adjacent to the effector genes, forming an effector–immunity (E–I) pair^19^.

The regulation of T6SS is controlled by a complex network influenced by both biotic and abiotic factors^20^, ^21^, ^22^, ^23^. Biotic factors, such as quorum‐sensing systems, two‐component systems, alternative sigma factors, and histone‐like proteins, modulate T6SS gene expression^22^, ^24^, ^25^. For instance, VasH, an alternative sigma factor encoded within the T6SS gene cluster, regulates T6SS gene expression in *Vibrio cholerae*, *Vibrio fischeri*, and *A. hydrophila* ^26^, ^27^, ^28^. Abiotic factors, including pH, temperature, oxygen levels, and iron availability, also play significant roles in regulating T6SS activity^20^, ^21^, ^29^. Temperature, in particular, is a critical signal for activating T6SS in marine bacteria^30^. Notably, the expression of Hcp, a key marker of T6SS activity, varies across these species at different temperatures, indicating that these bacteria utilize temperature‐dependent regulatory mechanisms to adapt to varying environmental and host conditions^21^, ^31^.

T6SS is widely present in *Aeromonas* species and plays a pivotal role in both inter‐bacterial competition and anti‐eukaryotic activity. For example, the lipase effector Tle1^AH^, encoded by *A. hydrophila* NJ‐35, contributes to biofilm formation and enhances in vivo fitness in crucian carp^6^. In *A. veronii* C4, the T6SS contributes to pathogenesis by disrupting host cell membrane integrity^32^, while *A. dhakensis* encodes several antibacterial T6SS effectors (TseI, TseP, and TseC) essential for its antibacterial activity^33^, ^34^, ^35^. Despite these findings, the functional characterization of T6SS in *A. hydrophila*, particularly in clinically isolated drug‐resistant strains, remains underexplored, especially regarding its role in virulence and competition in diverse host environments.

In this study, we characterized the genetic structure and functions of T6SS in *A. hydrophila* strain BJ054 (hereafter referred to as AH54), a drug‐resistant clinical isolate from the hydrothorax of a patient with esophageal cancer^3^. Comparative genomic analyses revealed that AH54 contains three distinct T6SS loci: a primary gene cluster and two homologous auxiliary clusters. We found that AH54 constitutively expresses T6SS, with activity notably at lower temperatures. Bioinformatics analysis identified four putative effector proteins (Rhs1, Rhs2, PAAR1, and PAAR2) encoded by the auxiliary clusters. These effectors show significant antibacterial activity, which is neutralized by corresponding immunity proteins. Notably, PAAR1 and PAAR2 are pivotal for Hcp secretion and contribute to the bactericidal capacity of T6SS. Beyond its role in bacterial competition, AH54's T6SS confers resistance to eukaryotic predation, especially by amoebae, via the delivery of a metal ion‐dependent DNase effector, Rhs2. Overall, our study provides a comprehensive characterization of T6SS in a clinically relevant *A. hydrophila* strain, advancing our understanding of T6SS diversity in *Aeromonas* species and providing valuable insights for future research on T6SS effector mechanisms in AH54.

## RESULTS

### Genetic characterization of T6SS gene loci in AH54

**Figure 1:**
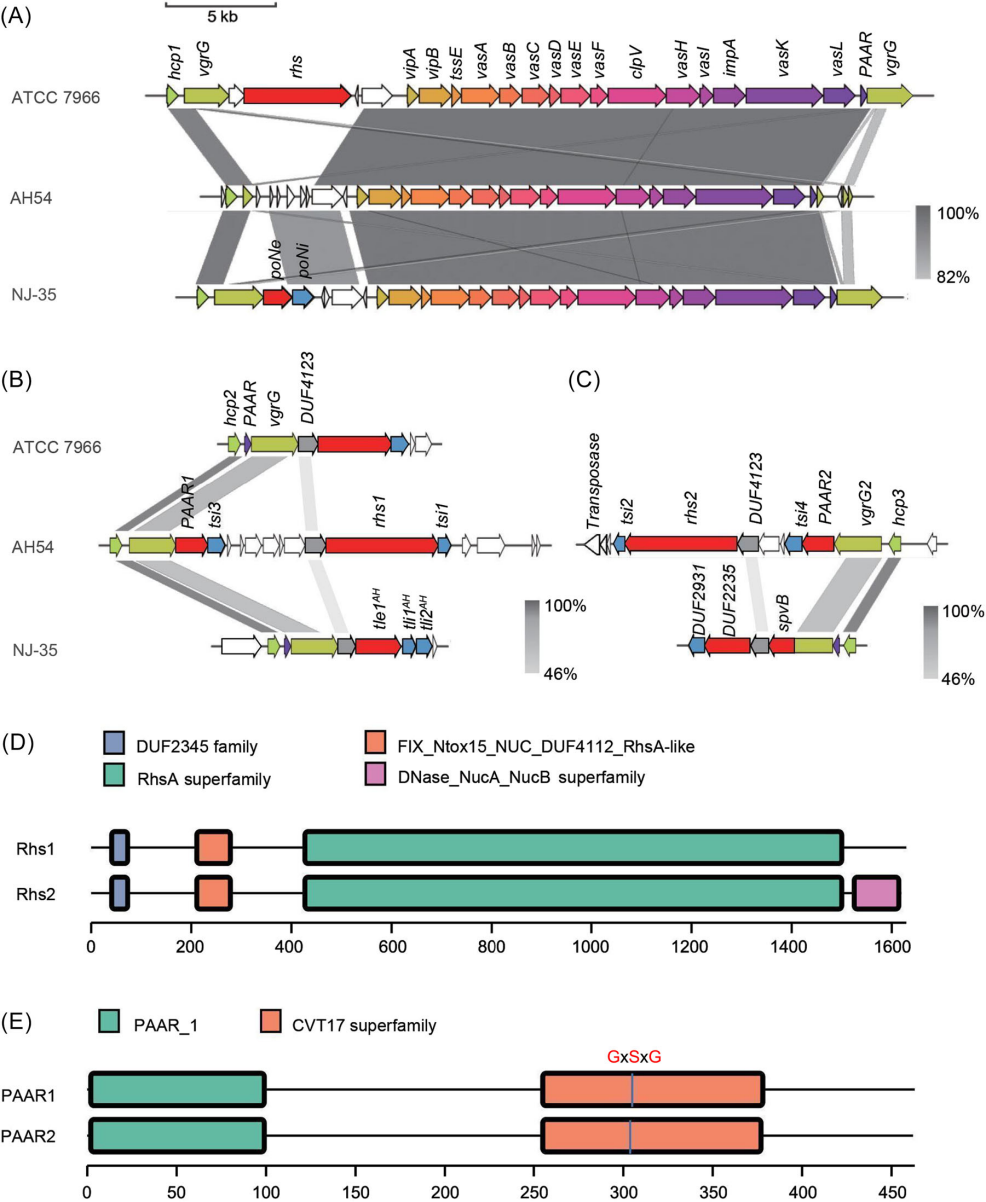
Genetic characterizations of T6SS gene clusters in *Aeromonas hydrophila* strains. (A–C) Comparative genomics analysis of the T6SS structural cluster (A), auxiliary cluster I (B) and cluster II (C) in *A. hydrophila* AH54, ATCC 7966, and NJ‐35. Four hypothetical effector–immunity (E–I) protein pairs from the *A. hydrophila* AH54 were identified and displayed. These E–I pairs are highlighted in red (effector) and blue (immunity). Sequence alignment was performed using BLASTn and the conserved sequence was visualized using EasyFig. (D, E) Schematic domain organization of T6SS effector proteins Rhs1/Rhs2 (D) and PAAR1/PAAR2 (E). Conserved domains were predicted using Batch CD‐Search.

*A. hydrophila* typically encodes a T6SS consisting of a structural gene cluster and one or more auxiliary gene clusters. However, the precise role of T6SS in clinical isolates of *A. hydrophila* remains largely unexplored. To identify T6SS‐associated proteins in the drug‐resistant clinical strain AH54, we performed a local BLASTP analysis using previously reported T6SS component protein sequences from *A. hydrophila* ATCC 7966 and NJ‐35^36^, ^37^. This analysis identified three T6SS genetic loci in AH54: one structural cluster and two auxiliary clusters (Aux1 and Aux2) (Figure 1A–C). All three strains share a conserved T6SS core cluster (Figure 1A); however, each auxiliary cluster in AH54 encodes two putative effectors, including rearrangement hotspot (Rhs) proteins and proline–alanine–arginine repeat (PAAR) effectors that are absent from the auxiliary clusters of the other two strains. Specifically, Rhs1 and PAAR1 are encoded by Aux1, while Rhs2 and PAAR2 are encoded by Aux2. This genetic organization contrasts with the reference genomes, which encode only one effector per auxiliary cluster (Figure 1B,C). Additionally, the core cluster of AH54 lacks an effector protein, Rhs in ATCC 7966 or PoNe in NJ‐35, unlike the reference genomes. To investigate whether these effector‐encoding genes are present in other *A. hydrophila* clinical isolates, PCR amplification using effector‐specific primers was performed on two additional clinical strains AH17 and AH18^3^. Amplified products for *rhs1*, *rhs2*, *PAAR1*, and *PAAR2* were detected in both AH17 and AH18 but not in the reference strain ATCC 7966 (Figure S1), suggesting that these clinical isolates may share common E–I protein modules and genetic organization.

We next used bioinformatics analysis to predict the structures of effectors encoded by AH54. The N‐termini of Rhs1 and Rhs2 were predicted to contain the FIX domain and DUF2345 (Figure 1D). The FIX domain, an intramolecular marker of T6SS effectors, has been previously reported^38^, and the core region includes a YD‐repeat (tyrosine–aspartate) motif, commonly associated with bacterial toxins^33^, ^39^. The C‐terminus of Rhs2 was predicted to belong to the NucA/B superfamily, indicating DNase activity^40^, while no functional domain was identified in the C‐terminus of Rhs1 using the Pfam database^41^ or other bioinformatics tools (Figure 1D), suggesting a novel function for Rhs1. PAAR1 and PAAR2 contain a PAAR domain and a C‐terminal toxic region, predicted to belong to the CVT17 superfamily (Figure 1E), with a conserved GxSxG motif potentially indicating phospholipase activity^6^, ^17^, ^42^. Phylogenetic analysis of T6SS lipase effectors placed both PAAR1 and PAAR2 in the Tle2 family (Figure S2). Since genes encoding immunity proteins are often located directly downstream of cognate effector genes, we designated Tsi1, Tsi2, Tsi3, and Tsi4 as the cognate immunity proteins for Rhs1, Rhs2, PAAR1, and PAAR2, respectively (Figure 1B,C). Together, these data suggest that AH54 encodes one complete T6SS structural cluster and two unique auxiliary clusters, each Aux with two E–I protein pairs.

### AH54 encodes an active T6SS against multiple competitors

**Figure 2:**
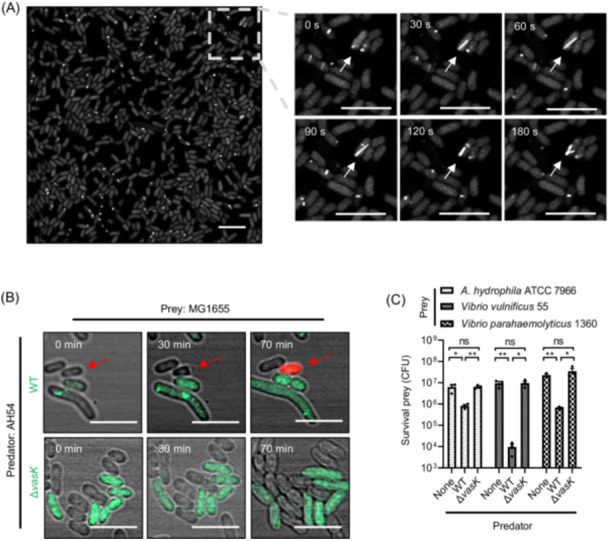
Functional characterizations of T6SS in *A. hydrophila* clinical strain AH54. (A) T6SS dynamics in AH54. VipA_sfGFP localization (sheath assembly) was monitored in at least 200 AH54 *vipA*_*sfGFP* cells by time lapse microscopy. Representative images of the GFP fluorescence channel are shown. Additional images with the time series of view can be found in Movie S1. The white arrow indicates the assembly and depolymerization of the T6SS during 180 s. Scale bar, 5 μm. (B) Competitive killing assay of predator AH54 and prey MG1655. AH54 *vipA*_*sfGFP* wild‐type (WT, upper) or its T6SS‐deficient mutant Δ*vasK* by knockout of an allelic T6SS gene (lower) was mixed with MG1655 at a 2 :1 ratio and blotted on a LB agar plate with propidium iodide (PI) dye at 28°C. Green cells indicate predator, cells without fluorescence represent prey, and red cells indicate dead prey cell during T6SS‐meidated competition (red arrow). A representative view from three similar results is displayed. The data of successive photos were related to Movies S2 and S3. Scale bar, 5 μm. (C) Bacterial competition assays between *A. hydrophila* and closely related species. Quantification of survival prey *A. hydrophila* ATCC 7966, *V. vulnificus* 55, and *V. parahaemolyticus* 1360 after a T6SS attack by predator AH54 WT and Δ*vasK*. The values represent mean ± SEM of three independent experiments. Statistical significance was determined using an unpaired Student's *t*‐test (ns, nonsignificant; *p < 0.05; **p < 0.01).

To assess whether AH54 constitutively expresses the T6SS, the AH54 *vipA*_sfGFP strain was constructed by replacing *vipA* on the chromosome with a *vipA_sfGFP* fusion via homologous recombination using the pRE112 suicide plasmid^43^, ^44^, ^45^. The cultures of the *vipA_sfGFP* strain, grown in LB medium at 28°C, were analyzed by microscopy imaging. As expected, most bacteria in the field of view showed active sheath assembly (Figure 2A; Movie S1). At the single‐cell level, we clearly observed the dynamic processes of initiation, assembly, extension, and contraction mediated by VipA_sfGFP (Figure 2A). These findings indicate that AH54 constitutively expresses T6SS under standard laboratory conditions.

To further investigate AH54's T6SS antibacterial activity, we performed bacterial competition assays via time‐lapse microscopy. Both predator AH54 *vipA_sfGFP* and prey *Escherichia coli* MG1655 were mixed and spotted onto an LB agar plate with propidium iodide (PI) dye. After approximately 30 min, *E. coli* MG1655 prey cells began to shrink, followed by the appearance of a red fluorescent signal upon T6SS‐mediated attack by AH54 (Figure 2B; Movie S2). In contrast, a T6SS‐deficient mutant (Δ*vasK*), which showed a similar growth rate to the wild type (WT) (Figure S3A), could not kill the prey cells (Figure 2B; Movie S3), confirming that T6SS in AH54 is responsible for antibacterial activity.

We next evaluated AH54's T6SS against related strain and species, including *A. hydrophila* ATCC 7966, *V. vulnificus* 55, and *V. parahaemolyticus* 1360, in bacterial competition experiments. Compared to the Δ*vasK* mutant, the AH54 WT strain significantly reduced prey survival (Figure 2C), suggesting that AH54 T6SS provides a competitive advantage over multiple competitors.

### Assembly dynamics, Hcp secretion, and antibacterial capacity of T6SS in AH54 are dependent on temperature changes

**Figure 3:**
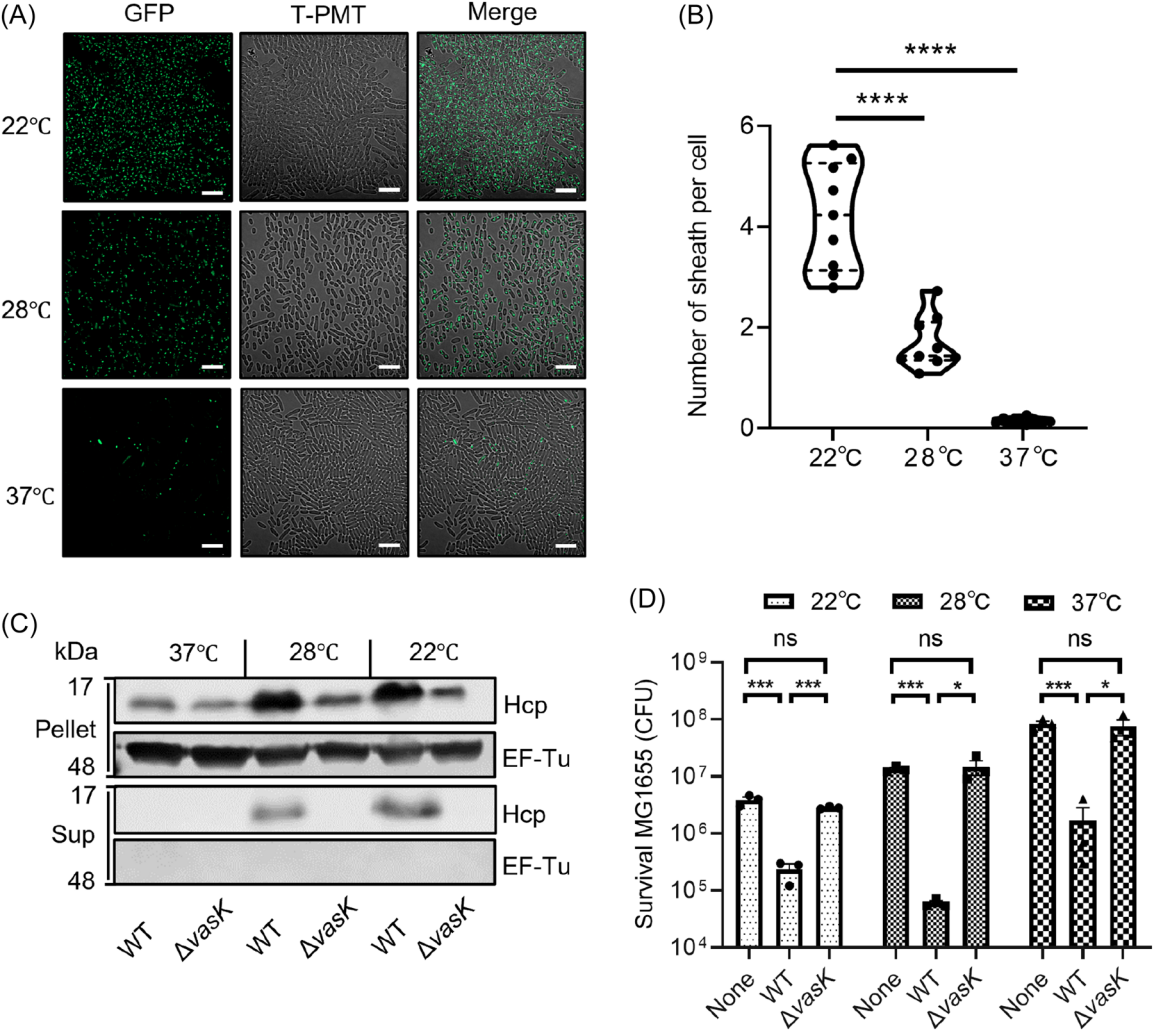
Temperature affects T6SS dynamics in AH54. (A) T6SS sheaths of AH54 *vipA*_*sfGFP* overnight cultures grown at 22°C, 28°C, and 37°C and monitored by confocal microscopy. Representative fluorescence micrographs from three independent replicates are shown. Scale bar, 5 μm. (B) Quantification of the sheath numbers per AH54 cell grown at 22°C, 28°C, and 37°C. The bold horizontal bar represents the median value; the bottom and top dash line of the internal boxplot correspond to the 25th and 75th percentiles, respectively. *p‐*values from all data were determined using an unpaired Student's *t*‐test, and statistical significance is indicated above the plots (ns, nonsignificant; ****p < 0.0001). (C) Western blot analysis of Hcp secretion in AH54 at different temperatures. AH54 was grown in LB medium to an OD_600_ of 1.5 at 22°C, 28°C, and 37°C. Cell pellets and 4 ml supernatants (Sup) were analyzed by SDS‐PAGE and immunoblot assays using anti‐Hcp and anti‐EF‐Tu primary antibodies. (D) Effect of temperature on AH54 T6SS‐mediated competition. A mixture of predator (AH54 WT or Δ*vasK*) and prey (MG1655) was grown in LB medium at indicated temperatures. After culturing for 4 h, the survival MG1655 (CFU) was determined using selective plates. The values represent mean ± SEM of three independent experiments. Statistical significance was determined using an unpaired Student's *t*‐test (ns, nonsignificant; *p < 0.05; **p < 0.01; ***p < 0.001).

To assess whether temperature affects the T6SS dynamics in AH54, AH54 *vipA_sfGFP* cells were grown at 22°C, 28°C, and 37°C, and sheath assembly was monitored using confocal microscopy. Results showed prominent sheath assembly at 22°C, moderate assembly at 28°C, and minimal activity at 37°C (Figure 3A). To quantify sheath assembly per cell at each temperature, over 4500 cells were analyzed. At 22°C and 28°C, cells assembled an average of 4 and 2 sheaths per cell, respectively, while at 37°C, sheath assembly nearly ceased, with only 0.17 sheaths per cell (Figure 3B). Subsequently, we detected expression levels of different T6SS relevant genes at different temperatures using qPCR. The expression levels of *vipA*, *vipB*, *vasH*, *hcp1*, *hcp2,* and *hcp3* were consistently higher at 22°C compared to both 28°C and 37°C (Figure S4A–F), and the same trend was observed for the expression of effector proteins of AH54 (Figure S4G,H). Interestingly, after the inactivation of T6SS of AH54 (Δ*vasK*), the expression levels of these genes were found to be significantly lower than that of WT at the same temperature (Figure S4).

Consistent with these observations, Hcp expression and secretion levels in AH54 cell pellets at 22°C and 28°C were significantly higher than a detectable level of Hcp at 37°C, indicating that temperature affects both Hcp expression and secretion (Figure 3C). Moreover, intracellular Hcp levels in AH54 Δ*vasK* cells at 22°C and 28°C were significantly lower than that in WT cells (Figure 3C). We further conducted bacterial competition assays to investigate the effect of temperature on antibacterial activity. AH54 efficiently killed prey cells at 22°C and 28°C, but bactericidal ability was reduced, though not abolished, at 37°C, because T6SS is able to secrete normally (Figures 3D and S5). Overall, these findings suggest that T6SS assembly dynamics, Hcp secretion, and antibacterial activity in AH54 are temperature‐dependent.

### AH54 encodes four functional antibacterial E–I protein pairs

**Figure 4:**
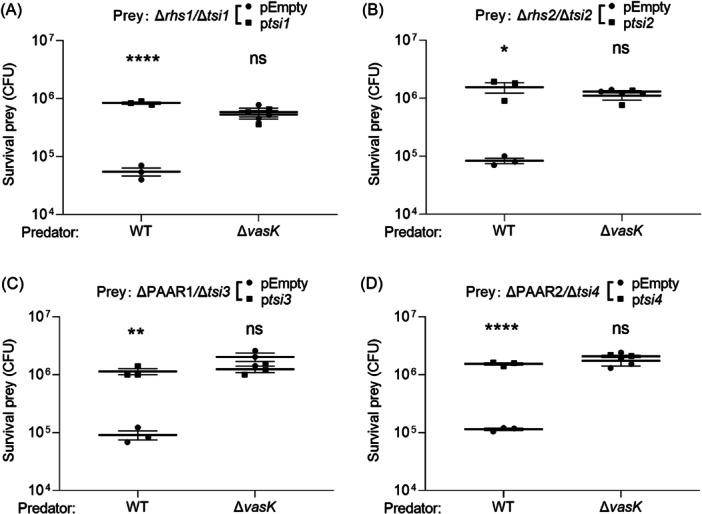
The effect of immunity proteins on neutralizing cognate effector toxicity. (A–D) Intra‐species competition assays between AH54 WT or Δ*vasK* mutants and Δ*rhs1*/Δ*tsi1* mutants (A), Δ*rhs2*/Δ*tsi2* mutants (B), Δ*PAAR1*/Δ*tsi3* mutants (C), Δ*PAAR2*/Δ*tsi4* mutants (D) are shown. Recovery of target cells of E–I mutants bearing with an empty plasmid (pSRKTc, pEmpty) or its derivative with the complementation of immunity (p*tsi1*, p*tsi2*, p*tsi3*, or p*tsi4*) after co‐cultured with WT and Δ*vasK* strains were investigated. The values represent mean ± SEM of three independent experiments. Statistical significance was determined using unpaired Student's *t*‐test (ns, nonsignificant; *p < 0.05; **p < 0.01; ****p < 0.0001).

To determine whether AH54 effector proteins’ antibacterial activity could be neutralized by their predicted cognate immunity proteins (Tsi1 to Tsi4), we generated sensitive mutants Δ*rhs1*/Δ*tsi1*, Δ*rhs2*/Δ*tsi2*, Δ*PAAR1*/Δ*tsi3*, and Δ*PAAR2*/Δ*tsi4* as prey and co‐incubated them individually with predator strains AH54 WT and Δ*vasK* to assess immunity protection. As expected, immunity‐deficient prey cells were sensitive to T6SS‐delivered cognate effectors, showing reduced survival when co‐incubated with the WT strain compared to Δ*vasK* (Figure 4A–D). However, complementing the corresponding immunity proteins in these strains restored resistance to WT killing, with survival levels comparable to Δ*vasK* (Figure 4A–D). This indicates that Rhs1/Tsi1, Rhs2/Tsi2, PAAR1/Tsi3, and PAAR2/Tsi4 function as T6SS antibacterial E–I pairs.

### PAAR1 and PAAR2 are required for Hcp secretion and effector delivery

**Figure 5:**
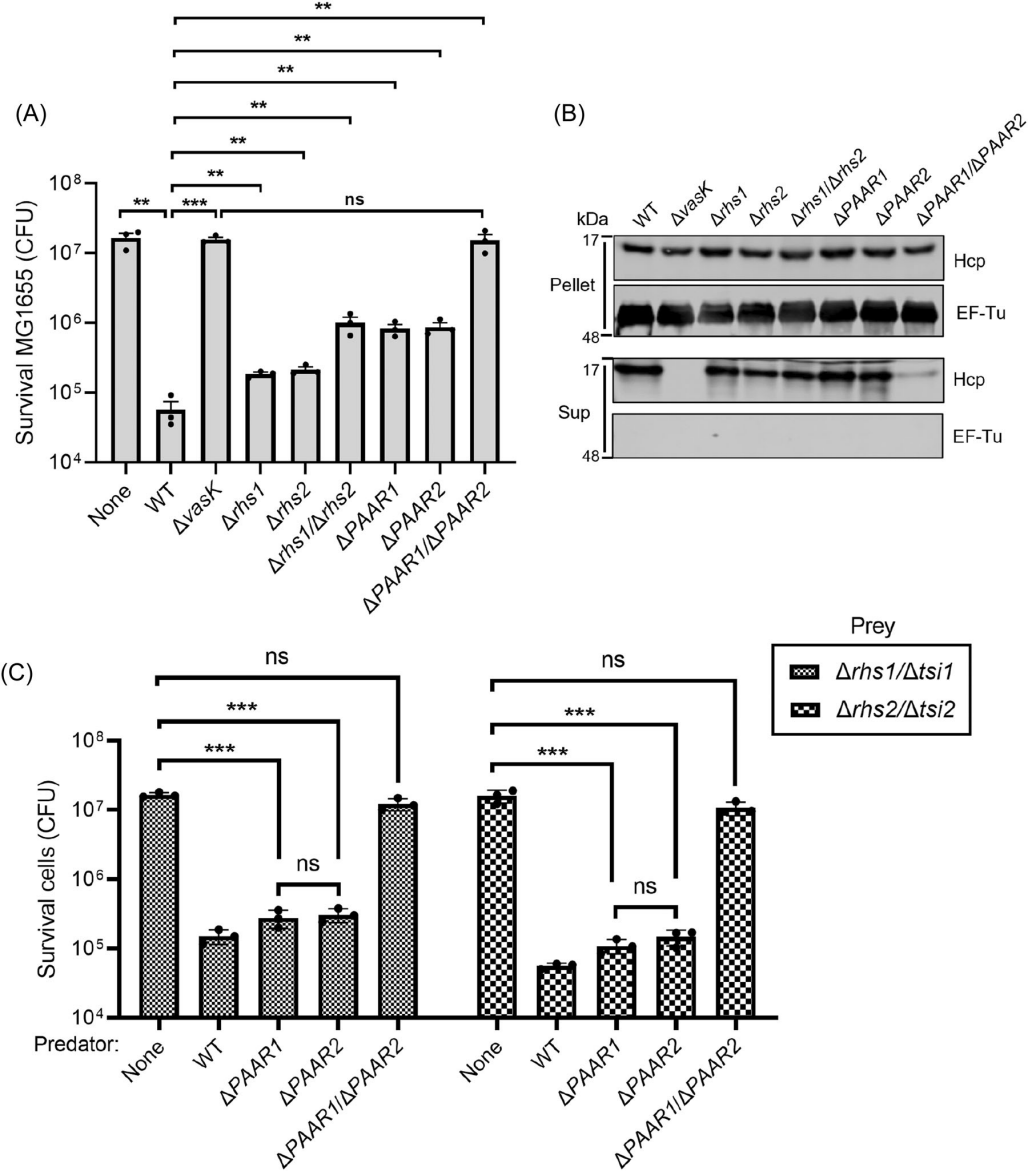
Effects of T6SS effector proteins of *A. hydrophila* AH54 on antibacterial ability and Hcp secretion. (A) Inter‐species competition assays between AH54 WT or other mutants and *E.coil* MG1655. Predator AH54 WT and its effector mutants were individually mixed with prey MG1655 at a 1:1 ratio and blotted on a LB agar plate at 28°C. After incubation for 4 h, the survival MG1655 (CFU) was determined using selective plates. The values represent mean ± SEM of three independent experiments. Statistical significance was determined using an unpaired Student's *t*‐test (*p < 0.05; **p < 0.01; ***p < 0.001; ****p < .0001). (B) The effect of effector on the T6SS assembly of *A. hydrophila* AH54. AH54 cells were grown to an OD_600_ of 1.5 at 28°C. Cell pellets and corresponding supernatants were analyzed by SDS‐PAGE and immunoblot assays using anti‐Hcp and anti‐EF‐Tu primary antibodies. (C) Bacterial killing assay between AH54 *PAAR* mutants and *rhs*/*tsi* mutants. The methodology is similar to (A). The values represent mean ± SEM of three independent experiments. Statistical significance was determined using an unpaired Student's *t*‐test (ns, nonsignificant; ***p < 0.001).

To characterize the antibacterial activity of AH54‐encoded effectors, we generated targeted deletion mutants and assessed their competitive capacity against *E. coli* MG1655 prey cells. The Δ*rhs1*, Δ*rhs2*, Δ*PAAR1*, and Δ*PAAR2* single mutants showed significantly reduced bactericidal activity compared to WT AH54 (Figure 5A), indicating that these effector genes are essential for full T6SS‐mediated antagonism. Strikingly, the Δ*PAAR1/*Δ*PAAR2* double mutant completely lost antimicrobial activity, phenocopying the T6SS‐deficient Δ*vasK* control (Figure 5A). This complete functional ablation suggests that the deletion of both PAAR1 and PAAR2 effectors abolished T6SS function even in the presence of the other two effectors.

Western blot analysis revealed similar levels of Hcp secretion between Δ*rhs1*, Δ*rhs2*, Δ*rhs1*/Δ*rhs2*, Δ*PAAR1*, and Δ*PAAR2* mutant strains and WT strains (Figure 5B). However, Hcp secretion in Δ*PAAR1*/Δ*PAAR2* double mutant was significantly less than the other mutant strains, showing partial inhibition rather than complete abrogation (Figure 5B). This partial secretion defect correlated with the complete loss of antibacterial activity, demonstrating that WT Hcp secretion levels require at least one functional PAAR protein.

Given the absence of PAAR domains in Rhs1/Rhs2 (Figure 1D), we investigated their delivery dependency on PAAR proteins through reciprocal competition assays. Single Δ*PAAR1* or Δ*PAAR2* mutants maintained effective killing of Δ*rhs1*/Δ*tsi1* and Δ*rhs2*/Δ*tsi2* targets (Figure 5C). Notably, the Δ*PAAR1*/Δ*PAAR2* double mutant failed to eliminate either target strain (Figure 5C), despite retaining partial Hcp secretion capacity (Figure 5B). This demonstrates that while individual PAAR proteins are dispensable for Rhs effector delivery, the presence of at least one PAAR homolog is strictly required for functional toxin translocation.

### Cognate immunity proteins can not provide cross‐protection against PAAR1 and PAAR2 effectors

**Figure 6:**
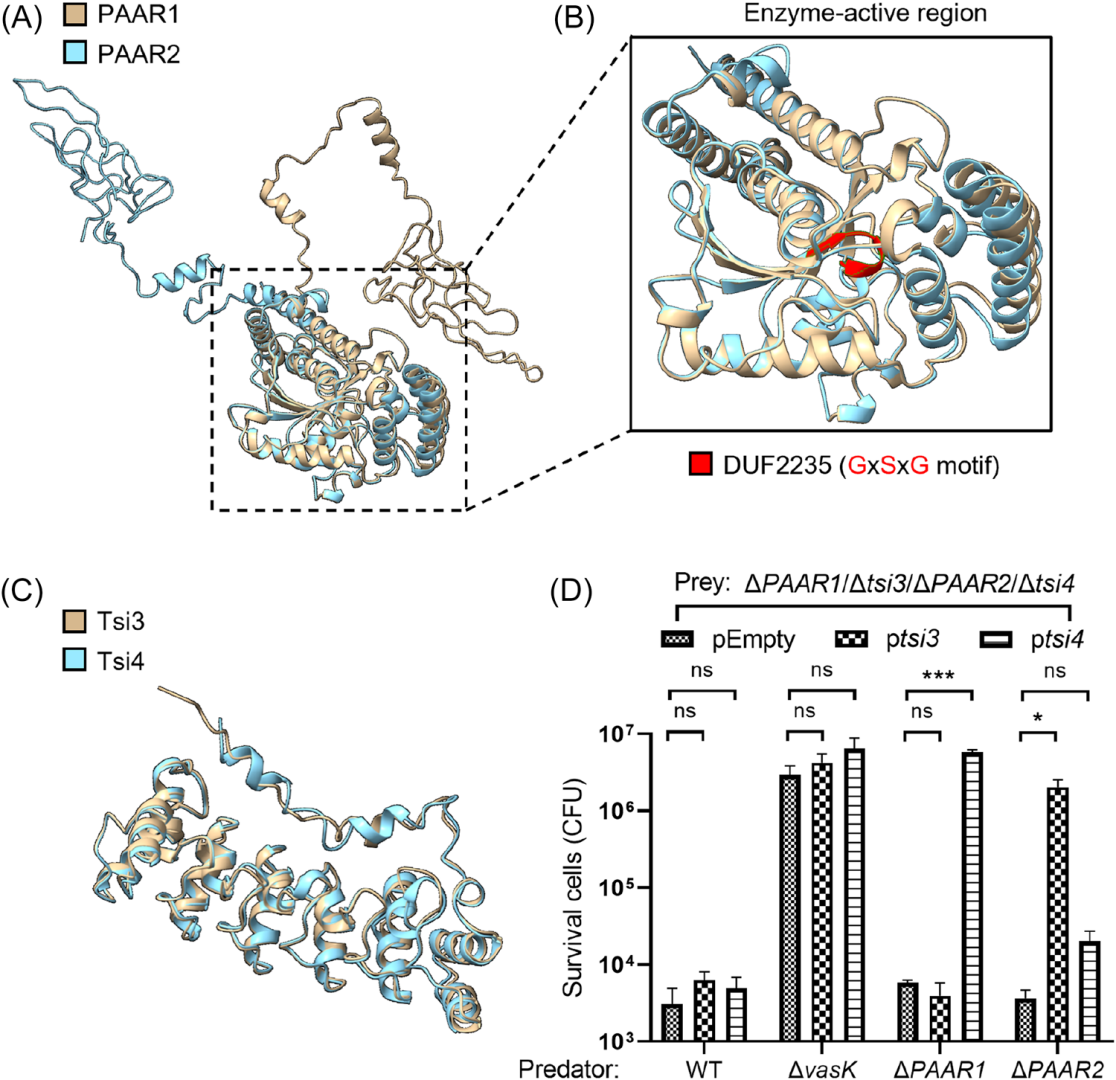
Effect of immunity proteins Tsi3 and Tsi4 on the toxicity of PAAR1 and PAAR2. (A) Alignments of the predicted structure of PAAR1 (golden) and PAAR2 (light blue) generated using AlphaFold 3.0. (B) Alignments of the enzyme‐active region of PAAR1 (golden) and PAAR2 (light blue) using Chimera X. The GxSxG motif for DUF2235 is highlighted in red. (C) Alignments of the predicted structure of Tsi3 (golden) and Tsi4 (light blue) generated using AlphaFold 3.0. (D) Quantification of survival prey Δ*PAAR1*/Δ*tsi3*/Δ*PAAR2*/Δ*tsi4* bearing a pSRKTc vector (pEmpty) or its derivatives with the complementation of immunity (p*tsi3* or p*tsi4*) after co‐cultured with predator AH54 WT, Δ*vasK*, Δ*PAAR1*, or Δ*PAAR2*. The values represent mean ± SEM of three independent experiments. Statistical significance was determined using an unpaired Student's *t*‐test (ns, nonsignificant; *p < 0.05; ***p < 0.001).

Effector proteins PAAR1 and PAAR2 share 93.94% sequence identity and show structural similarity, as predicted by AlphaFold 3.0 analysis (Figures 6A and S6A). It was determined that both PAAR1 and PAAR2 possess the conical cap structure. We then superimposed PAAR1 and PAAR2 protein structures; the result showed that the PAAR structural domains of PAAR1 and PAAR2 were identical (Figure 6A). Another part of PAAR1 and PAAR2 structures, the potential enzyme‐active region, has the same GxSxG motif, and except for several differences in amino acid sequence alignment, the rest of the structures were not much different (Figure 6B). Similarly, their cognate immunity proteins, Tsi3 and Tsi4, display 79.46% sequence identity and structural resemblance (Figure S6B,C). The protein structures of Tsi3 and Tsi4 are mainly different in the N‐terminal; Tsi3 has a random coil and Tsi4 has an α‐helix (Figure 6C).

To demonstrate whether PAAR1 and PAAR2 could be neutralized by each other's immune proteins, we first predicted the interaction between PAAR1 and Tsi3 and the interaction between PAAR2 and Tsi4 through AlphaFold 3.0. The superimposed interaction result showed a high degree of overlap between two effector proteins at the site of interaction with their own immune proteins (Figure S6C). Then, we examined the cross‐protection of non‐cognate immunity proteins against PAAR1 and PAAR2 using bacterial competition assays. The results showed that complementation of Tsi3 (for PAAR1) in the Δ*PAAR1*/Δ*tsi3*/Δ*PAAR2*/Δ*tsi4* mutant did not neutralize PAAR2‐mediated toxicity from the WT or Δ*PAAR1* strain (Figure 6D). Similarly, complementation of Tsi4 (for PAAR2) did not protect against PAAR1‐mediated toxicity from the WT or Δ*PAAR2* strain (Figure 6D). To address this issue, we analyzed the interacting region of effector and immunity proteins and found that they differ from each other due to differences of individual amino acids (Figure S6D).

### Rhs2 is a DNase toxin with metal ion‐dependent activity

**Figure 7:**
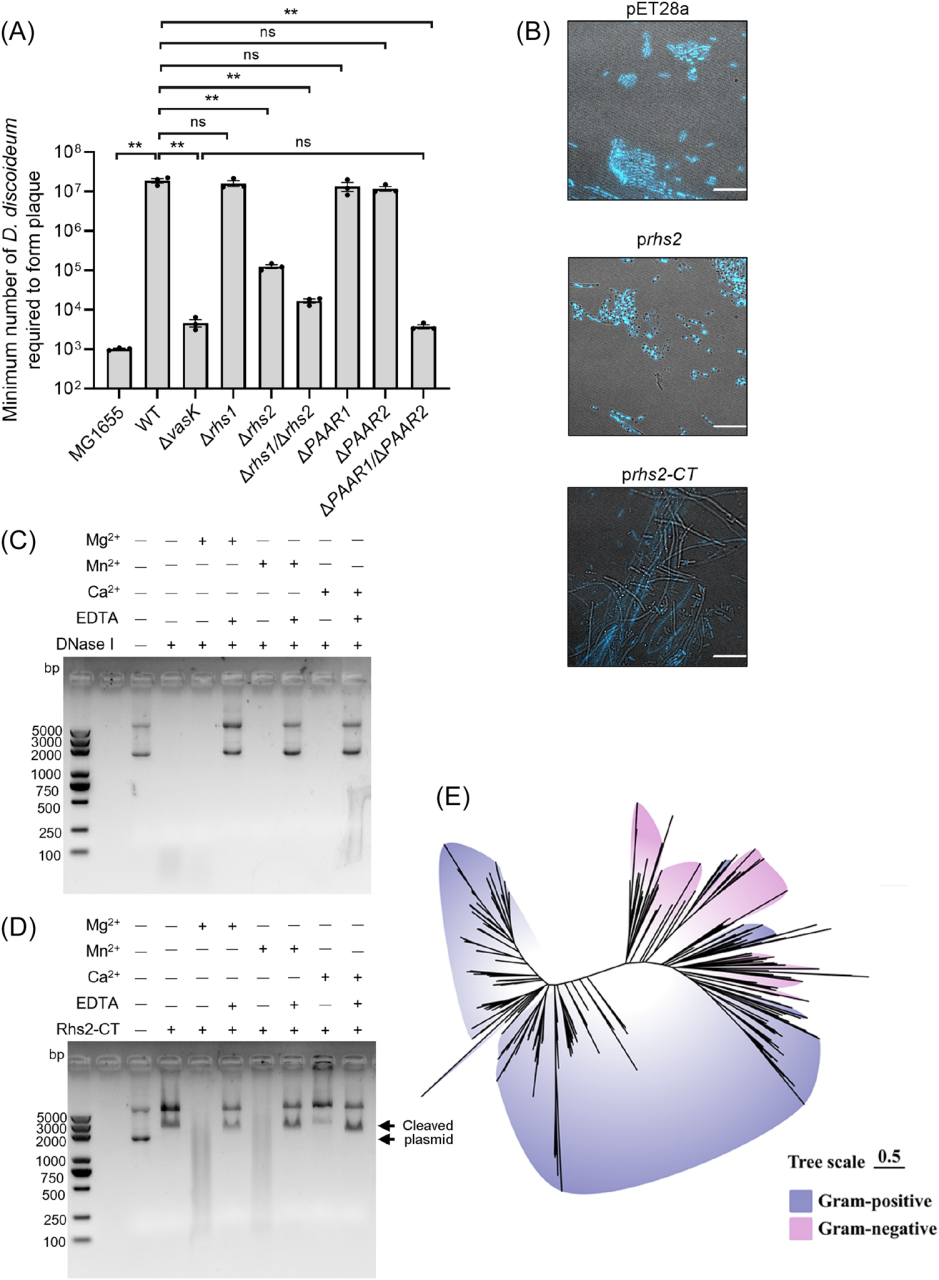
T6SS mediates AH54's virulence on eukaryotic cells. (A) Virulence of *A. hydrophila* AH54 T6SS toward *Dictyostelium discoideum*. Plaque assay was performed to compare strains AH54 WT, Δ*vasK*, Δ*rhs1*, Δ*rhs2*, Δ*rhs1/*Δ*rhs2*, Δ*PAAR1*, Δ*PAAR2*, and Δ*PAAR1/*Δ*PAAR2*. *D. discoideum* was serially diluted on nutrient SM/5 agar inoculated with indicated bacteria and incubated at 22°C for 4 days to allow for plaque formation. The values represent mean ± SEM of three independent experiments. Statistical significance was determined using an unpaired Student's *t*‐test (ns, nonsignificant; **p < 0.01). (B) Effect of expression of Rhs2 and Rhs2‐CT on intracellular DNA digestion. Fluorescence microscopy images of *Escherichia coli* BL21(DE3) cells harboring pET28a or its derivatives of p*rhs2* or p*rhs2‐CT* after IPTG induction and DAPI staining are shown. Scale bars, 10 μm. (C, D) Metal ion‐dependent DNase activity assay of Rhs2‐CT. Plasmid pUC19 was incubated with Rhs2‐CT or DNase I in reaction buffer with or without Mg^2+^, Ca^2+^, and Mn^2+^ at 37°C for 1 h. Reaction products were analyzed using agarose gel electrophoresis. (E) Maximum‐likelihood phylogeny of Rhs2‐CT proteins. The light purple and pink shadows represent proteins from Gram‐positive bacteria and Gram‐negative bacteria, respectively.

Studies have shown that bacteria use cytotoxic T6SS effectors to counteract eukaryote phagocytosis in the *Dictyostelium discoideum* (Dicty) model^7^, ^34^, ^46^. When anti‐eukaryotic toxins are secreted, more Dicty cells are required to form plaques on a bacterial lawn. We evaluated the role of AH54 T6SS in eukaryotic cell predation and found that significantly more Dicty cells were needed to form plaques on the WT AH54 lawn compared to *E. coli* MG1655 and the Δ*vasK* mutant, indicating that AH54 T6SS is a key virulence factor against Dicty predation (Figure 7A). We then evaluated whether AH54 antibacterial proteins contribute to T6SS‐dependent anti‐amoeba activity. The minimum Dicty cell count required for plaque formation on Δ*rhs2* and Δ*rhs1*/Δ*rhs2* lawns was substantially lower than that for WT, Δ*rhs1*, Δ*PAAR1*, and Δ*PAAR2* strains, demonstrating that Rhs2 is crucial for amoeba predation resistance (Figure 7A). Interestingly, the Dicty cell count required to form plaques on the Δ*PAAR1*/Δ*PAAR2* lawn was similar to that on the Δ*vasK* lawn because the Δ*PAAR1*/Δ*PAAR2* mutant lost the delivery capacity of Rhs proteins. These findings suggest that T6SS‐encoded Rhs2 directly contributes to the eukaryotic virulence of AH54.

Given the capacity of Rhs2 to target both prokaryotic and eukaryotic cells, we further investigated its mechanism of action. To assess whether Rhs2 shows DNase activity, as previously predicted, we performed an in vivo DNase assay^15^, ^47^. *E. coli* BL21(DE3) cells expressing either full‐length Rhs2 or truncated Rhs2‐CT were induced with IPTG and stained with DAPI. Fluorescence microscopy revealed that most of the BL21(DE3) cells expressing Rhs2 or Rhs2‐CT lost DAPI staining after 4 h of induction, in contrast to cells carrying the pET28a vector (Figure 7B), indicating DNase activity in vivo. Notably, Rhs2‐CT expression induced pronounced filamentation in BL21(DE3), manifested as elongated cellular morphology with defective septation (Figure 7B), suggesting cell division impairment secondary to genotoxic stress.

To directly verify DNase activity of Rhs2, we purified Rhs2‐CT proteins (Figure S7A,B) and performed an in vitro DNase activity assay. The addition of Rhs2‐CT proteins led to partial plasmid degradation with linearized products compared to the plasmid‐only control (Figure 7C,D). To evaluate the effect of metal ions on the DNase activity of Rhs2‐CT, we measured enzymatic activity in the presence of Mn²⁺, Mg²⁺, Ca²⁺, and EDTA, an inhibitor of ion‐dependent nucleases. Compared to the control of Rhs2‐CT alone, the plasmid was fully digested only in the presence of Mn²⁺ or Mg²⁺, but not Ca²⁺. Moreover, the addition of excess EDTA completely inhibited Mn²⁺‐ or Mg²⁺‐dependent activity, resembling the plasmid‐only control (Figure 7D). These findings indicate that Rhs2's enzymatic activity requires Mn²⁺ or Mg²⁺.

Subsequently, we systematically analyzed the phylogenetic distribution of Rhs2‐CT domain homologs across bacterial taxa. A BLASTp homology search using Rhs2‐CT protein sequences as queries yielded 456 nonredundant hits (*E* value < 2e^−63^), as detailed in Supporting Information–Data S2. Notably, Gram‐positive bacteria predominated (78.07%) within this dataset. Given that Gram‐positive species typically lack canonical T6SS components, this phylogenetic bias implies that Rhs2‐CT domains may primarily function in biological contexts distinct from T6SS‐mediated processes (Figure 7E). Consistent with this hypothesis, bioinformatic analyses revealed that Rhs2‐CT‐containing proteins showed either autonomous secretion signals or functional associations with the alternative secretion machinery, including but not limited to Type VII Secretion System (T7SS) components (Supporting Information–Data S2). Collectively, these findings support a model wherein Rhs2‐CT domains likely operate through mechanistically diverse pathways, thereby potentiating bacterial environmental adaptation and pathogenicity enhancement.

## DISCUSSION

*A. hydrophila* encounters a wide variety of competitors in both natural and host environments. To gain a competitive edge and dominate ecological niches, this pathogen has developed several defensive strategies, including the T6SS, which enables it to kill or inhibit rival organisms^6^, ^28^. This study focused on the T6SS gene cluster in clinical isolates of *A. hydrophila*, specifically the multi‐drug‐resistant strain AH54. While T6SS‐mediated antagonism has been characterized in *A. hydrophila*, its function in clinical strains remains poorly understood^6^, ^48^. Here, we present the identification of a complete T6SS apparatus in AH54, comprising one structural cluster and two auxiliary clusters encoding distinct E–I protein pairs: Rhs1/Tsi1 and PAAR1/Tsi3 in Aux1 and Rhs2/Tsi2 and PAAR2/Tsi4 in Aux2. Although PAAR1 and PAAR2 share high homology, their respective immunity proteins do not offer cross‐protection, suggesting that each PAAR protein plays a unique role. We also observed that T6SS activity in AH54 is temperature‐dependent and influenced by effector proteins such as PAAR1 and PAAR2. Notably, Rhs2 acts as a DNase toxin with dual targets, showing activity against both eukaryotic and prokaryotic competitors.

In complex microbial communities, T6SS is a powerful tool commonly used by Gram‐negative bacteria to mediate antagonistic interactions among different bacterial species^16^, ^17^. Using time‐lapse confocal imaging, we observed the dynamics of T6SS in AH54 T6SS, including initiation, assembly, extension, and contraction (Figure 2A). When the prey cell was punctured by the T6SS of WT AH54, membrane compromise and subsequent cell death were clearly visible, confirming that the AH54 T6SS is functional (Figure 2B; Movie S2). In ecological niches such as the host or aqueous environments, when AH54 uses T6SS to eliminate competing bacteria, the DNA of these killed cells is released and dispersed as mobile genetic elements (MGEs), including resistance and virulence genes. Through horizontal gene transfer, AH54 can acquire these genes, thereby enhancing its competitive advantage and virulence^49^. We speculate that T6SS of AH54 and closely related clinical strains might play an important role in eukaryotic virulence.

We found that AH54 shows robust T6SS assembly dynamics and antimicrobial activity at lower temperatures, with a significant decrease in T6SS activity at 37°C (Figure 3A–C). Bacterial competition assays demonstrated that WT AH54 retained the ability to kill *E. coli* MG1655 prey cells at 37°C, as T6SS maintained secretory activity, though at reduced levels (Figures 3D and S5). While this finding may seem contradictory, given AH54's clinical nature, it highlights the high energy demands of T6SS and its critical role in inter‐bacterial competition. Therefore, regulating T6SS activity under different environmental conditions is important^8^, ^31^, ^50^. AH54, isolated from the hydrothorax of a patient with esophageal cancer and lacking polymicrobial infection^3^, may not need to compete with other bacteria in this environment. Thus, AH54 may downregulate T6SS activity at 37°C to conserve energy for other virulent factors. Moreover, sources of AH54 infection originate from contaminated seafood^3^, and AH54 T6SS has killing ability against bacteria and anti‐predation activity against amoeba cells (Figures 3D and 7A), which widely exist in natural environments^30^, ^51^. This temperature‐dependent regulation of T6SS allows AH54 to adapt to diverse environments, balancing competition with energy conservation^21^, ^30^. In natural, low‐temperature environments, AH54 actively uses T6SS to eliminate competitors, while in the host, it reduces T6SS activity without fully silencing it, preserving energy for other virulence factors and maintaining a competitive advantage in polymicrobial infections.

We confirmed that AH54 encodes four functional antibacterial effectors, with PAAR1 and PAAR2 being essential for Hcp secretion, in line with their role in initiating T6SS assembly. In contrast, Rhs1 and Rsh2, while non‐essential for Hcp secretion, show potent antibacterial activity (Figure 5A,B). Interestingly, the deletion of both PAAR1 and PAAR2 resulted in a loss of antimicrobial capacity, even though T6SS secretory activity remained partly intact, suggesting that PAAR1 and PAAR2 are crucial for the assembly of T6SS and the delivery of Rhs1 and Rhs2 (Figure 5A–C). This implies a potential interaction between these PAAR and Rhs effectors. Despite their high sequence identity and structural similarity, PAAR1 and PAAR2 show nonredundant functions (Figure 6A–C). The lack of cross‐protection between their cognate immunity proteins, Tsi3 and Tsi4, further supports the idea that each PAAR effector uniquely contributes to AH54's competitive fitness. Additionally, while the interaction patterns between PAARs and their immunity proteins are similar (Figure S6C), subtle differences in amino acids involved in these interactions could explain their distinct roles (Figure S6D). For instance, Jiang et al. found that a key tyrosine residue (Tyr119) in *B. fragilis* peptidyl‐prolyl isomerase is critical for ubiquitin homologue sensitivity, with strains encoding a glutamate at this position being resistant^52^. This suggests that variations in the physicochemical properties of specific amino acids could lead to distinct neutralizing effects in the PAAR‐immunity system.

Rhs proteins are characterized by a conserved N‐terminal delivery domain and a variable C‐terminal domain with diverse toxic functions^33^, ^53^, ^54^, ^55^. The C‐terminal domains of Rsh1 and Rhs2 are critical for their toxicity, with Rhs2 being identified as a metal ion‐dependent DNase, and the C‐terminus of Rhs1 encoding a novel, yet‐to‐be‐defined domain (Figure 1D). Both PAAR1 and PAAR2 have an N‐terminal PAAR domain and a predicted C‐terminal DUF2235 domain with a catalytic GxSxG motif (Figure 1E), which is commonly associated with phospholipase activity^6^, ^17^, ^42^. These two effectors are also predicted to belong to the Tle2 lipase family (Figure S2), which has been found to show eukaryotic toxicity^42^, ^56^. Therefore, further investigation is necessary for elucidating its antibacterial mechanism. Recent studies have highlighted the role of T6SS effectors in manipulating host cells and enhancing bacterial virulence^32^, ^57^. In *Aeromonas* species, T6SS has demonstrated functionality in eukaryotic cell targeting^14^, though specific mechanisms remain unclear. In AH54, T6SS plays a critical role in defense against eukaryotic predators, as evidenced in the *D. discoideum* model. Rhs2 was identified as a key contributor to eukaryotic toxicity (Figure 7A). While the mechanism for Rhs2 nuclear translocation is not fully understood, research on the T6SS DNase effector TafE in *Acinetobacter baumannii* suggests that bacterial toxins may use unconventional nuclear localization signals for translocation^58^. Furthermore, the dual functionality of Rhs2 highlights the evolutionary adaptability of T6SS effectors, allowing them to meet competitive pressures in both microbial and host environments, thereby enhancing our understanding of the multifunctional nature of these effectors.

In summary, this study provides a comprehensive characterization of the T6SS genetic architecture in drug‐resistant *A. hydrophila* strain AH54, with a particular focus on the unique effector combination, especially the dual‐function Rhs2. Our findings position AH54 as a valuable model for studying the balance between interbacterial competition and anti‐eukaryotic virulence. These insights contribute to a deeper understanding of T6SS diversity in clinical *A. hydrophila* isolates and their complex interactions with other organisms. The knowledge gained from this study could inform the development of new antimicrobial strategies aimed at combating drug‐resistant bacterial infections.

## MATERIALS AND METHODS

### Bacterial strains and growth conditions

All strains and plasmids used in this study are listed in Tables S1 and S2, (Supporting information‐Data S1) respectively. Strains were cultured at 22°C, 28°C, or 37°C in Luria–Bertani medium or on 1.5% agar plates. The final antibiotic concentrations in the growth medium were as follows: 10 μg/ml chloramphenicol (Cm), 100 μg/ml ampicillin (Amp), 100 μg/ml streptomycin (Str), and 10 μg/ml tetracycline (Tc).

### Genetic construction in *A. hydrophila*

The primers used in this study are listed in Table S3. Gene deletions and knock‐ins were constructed using the suicide plasmid pRE112, which contains a *sacB* counter‐selectable marker as previously described^43^. Briefly, 600‐bp upstream and 600‐bp downstream fragments of the target gene were amplified, and the PCR products were ligated into a pRE112 vector digested with *Xba*I and *Kpn*I restriction enzymes using Gibson assembly. Double‐crossover cells were selected on 10% sucrose LB agar plates without salt. Deletion or insertion mutants were confirmed by PCR and sequencing. For complementation assays, the *tsi1‐* to *tsi4*‐encoding genes were cloned into an IPTG‐inducible pSRKTc plasmid to induce immunity protein expression^59^. For protein purification, the C‐terminal extension domain of *rhs2* (*rhs2‐CT*) was cloned into a pET28a plasmid with a 6×His tag and transformed into *E. coli* BL21(DE3).

### Bacterial competition assays

Overnight cultures of bacterial predators and prey cells were diluted to OD_600_ of 1.0 and mixed at a 1:1 (predator: prey) ratio. A 5 μl aliquot of the mixture was spotted onto a 0.22 μm polyvinylidene fluoride (PVDF) membrane placed on an LB agar plate and incubated at 28°C. After 4 h, the cells were collected in 1 ml of 0.8% NaCl and 5 μl of serial dilutions were spotted onto selective LB agar plates to determine the number of surviving prey cells. When necessary, 0.1 mM IPTG was added to the solid medium.

### Measurement of the growth curve

Overnight cultures were diluted 1:100 into fresh LB media and incubated at 28°C with shaking at 220 rpm. The OD_600_ was measured every 60 min for each strain.

### RT‐qPCR analysis

Bacterial cultures grown in LB medium were harvested and subjected to total RNA isolation using TRIZOL reagent. Single‐stranded cDNA was conducted with 600 ng of total RNA as template in 10 μl reactions containing HiScript® III RT SuperMix (Vazyme R323) and random hexamer primers. The resultant cDNA products were normalized to a 20 ng/μl working concentration for downstream analysis. Reverse transcription‐quantitative PCR (RT‐qPCR) was performed on a LightCycler® 96 SW system (Roche Diagnostics) with gene‐specific primers (see Table S3). To standardize the results, the relative abundance of the *A. hydrophila* 16S gene was used as the internal standard. The fold change in gene transcription was determined using the comparative threshold cycle (CT) method.

### Protein secretion and Western blot analysis

Overnight bacterial cultures were diluted 1:100 into fresh LB medium and incubated at specified temperatures until reaching OD_600_ of 1.5. The cultures were centrifuged (3000*g*, 5 min, 4°C), and the supernatant was filtered through a 0.22 μm PVDF filter. For protein precipitation, culture supernatants were acidified with 100% trichloroacetic acid at a 10% (v/v) final concentration (200 μl additive per 1.8 ml supernatant). The acidified mixtures were incubated for 2 h on ice before high‐speed centrifugation (20,000*g*, 15 min, 4°C). Protein pellets were subsequently subjected to solvent washing using pre‐chilled acetone (−20°C) with equivalent volume. Protein samples were separated on a 12% SDS‐PAGE gel and transferred onto a PVDF membrane, which was blocked with 5% nonfat milk. The PVDF membrane was probed overnight at 4°C with the primary antibodies: anti‐Rabbit Hcp, anti‐Mouse EF‐Tu, and anti‐Mouse RpoB. It was then incubated with fluorescent secondary antibodies under light‐avoided conditions by wrapping the antibody incubation box with aluminum foil. The signal was screened using a two‐color infrared laser imager.

### Fluorescence microscopy

For quantification of T6SS activity, cells grown at various temperatures were resuspended in LB and spotted on 1.5% agarose‐LB pads. All microscopy images were acquired using a ZEISS LSM900 inverted microscope equipped with Airyscan and Definite Focus 2.0 systems, capturing images through the GFP channel. Each sample was observed continuously for 10 min, with images taken every 30 s or at specific intervals depending on the experimental objective. For inter‐bacterial competition experiments, overnight cultures were washed once with LB, diluted 1:100 into fresh medium supplemented with the appropriate antibiotics, and grown to OD_600_ of 1.0. Cells from 1 ml of the culture were concentrated 10‐fold, mixed at a 2:1 ratio (predator to prey), spotted onto a thin 1.5% agarose pad containing fluorescence dye (PI), and then covered with a glass coverslip. Bacterial interactions were imaged over 2 h at 28°C using a ZEISS LSM900 inverted microscope with Airyscan and Definite Focus 2.0 systems. Images were captured every 60 s.

### Image analysis

Fiji was used for all image analyses and manipulations^60^. Cell numbers in each fluorescence image were quantified using two methods: an automated cell counting algorithm and manual counting. For the automated approach, the fluorescence image threshold was adjusted to ensure accurate cell counting. The number of cells was then determined using the Analyze Particles function, with parameters adjusted to match cell size and to minimize discrepancies between automated and manual counts. All automated results were subsequently verified through manual counting. For quantifying T6SS activity, the fluorescence image threshold was adjusted to include all T6SS sheaths, and the fluorescence spots were counted using the Analyze Particles function in Fiji. Manual counting was then performed to verify the T6SS sheath counts. All imaging experiments were performed in triplicate with at least three biological replicates.

### Bioinformatics analysis

The complete genome sequence of *A. hydrophila* AH54 was obtained through whole‐genome sequencing (Magigene). Gene sequences of *A. hydrophila* ATCC 7966 and NJ‐35 were retrieved from the full‐length genome assemblies (GenBank accession numbers: NC_008570.1 and NZ_CP006870.1). Comparative genomic analysis of the T6SS gene clusters was conducted using EasyFig v2.2.2^61^. The protein sequences of Rhs1, Rhs2, PAAR1, and PAAR2 were analyzed using the Conserved Domain Database (CDD)^62^.

### *D. discoideum* plaque assays

The phagocytosis amoeba model was used to evaluate T6SS‐mediated anti‐eukaryotic activity, as previously described^7^, ^34^, ^51^, ^63^. Briefly, *A. hydrophila* AH54 and *E. coli* MG1655 cells were grown overnight at 37°C for 12 h, pelleted by centrifugation at 5000 rpm for 5 min, and resuspended in phosphate‐buffered saline (PBS) to OD_600_ of 1.0. A 100 μl aliquot of bacterial suspension was spread onto SM/5 plates and allowed to dry at room temperature. *D. discoideum* cells (~2.5 × 10^8^ cells/ml) were harvested after 3 days of growth on SM plates, pelleted by centrifugation at 500*g* for 2 min, washed three times with PBS, and resuspended in 1 ml of PBS. A 10‐fold serial dilution of *D. discoideum* cells was plated onto the prepared SM/5 plates. Plaques were counted after 4 days of incubation at 22°C. The minimum number of *D. discoideum* cells required to form a plaque on the bacterial lawn was recorded.

### DAPI staining analysis

To perform DAPI staining analysis^15^, ^47^, overnight cultures of *E. coli* BL21(DE3) containing the pET28a plasmid or its derivatives, pET28a‐*rhs2* and pET28a‐*rhs2‐CT*, were diluted 100‐fold in LB broth and incubated at 37°C with shaking at 220 rpm. Toxin expression was induced by the addition of 0.15 mM IPTG, followed by 4 h of incubation at 28°C. Cells were collected, resuspended in PBS with 1% Triton X‐100 for 5 min, and then incubated in PBS with 10 μg/ml DAPI at room temperature for 10 min. Cells were visualized using a confocal laser scanning microscope (ZEISS LSM900).

### Expression and purification of proteins

Overnight cultures containing pET28a‐*rhs2*‐*CT* were inoculated into LB medium and grown to an OD_600_ of 1.0. Protein expression was induced with 0.15 mM IPTG for 6 h at 37°C. Cells were collected by centrifugation and resuspended in loading/washing buffer (50 mM HEPEPS, 300 mM NaCl, 30 mM imidazole, pH 7.8). The cells were lysed using a cell disruptor at 4°C, and the lysate was clarified by centrifugation at 18,000*g* for 30 min. Recombinant proteins with a 6xHis tag was purified using a Ni²⁺‐NTA column (Sangon Biotech). The column was washed with 100 ml of loading/washing buffer and eluted with elution buffer (50 mM HEPES, 300 mM NaCl, 400 mM imidazole, pH 7.8). The eluted proteins were further purified using the AKTA FPLC system.

### DNase activity assays

Purified Rhs2‐CT protein (0.04 nmol) or DNase I (5 U) was incubated with pUC19 (400 ng) in reaction buffer (0.15 mM NaCl, pH 7.0). Where indicated, 2 mM MnCl_2_, MgCl_2_, CaCl_2_, or 10 mM EDTA were added. The DNA hydrolysis reaction was performed at 37°C for 1 h, and DNA integrity was analyzed by 0.8% agarose gel electrophoresis.

### Construction of a phylogenetic tree

Representative members of the Tle1‐4 family protein sequences were obtained as previously reported^6^. Multiple sequence alignments were performed using CLUSTALW, and phylogenetic trees were constructed using MEGA X software using maximum likelihood with 1000 bootstrap replicates^64^. The phylogenetic trees were visualized using the ChiPlot ^65^ online platforms.

A BLASTp search in the refseq_select_prot database was performed using the C‐terminal sequence of Rhs2 (A1501‐K1619) as the query. Homologous protein sequences were obtained with an E‐value threshold less than 0.05 and at least 26.47% identity. Using MAFFT (v7.310)^66^, multiple sequence alignment was performed on these homologous protein sequences. IQ‐TREE (v2.0.3)^67^ was then used to construct a maximum likelihood phylogenetic tree, which was subsequently edited and annotated using ChiPlot^65^ online platforms. Species information from the BacDive database^68^ was retrieved to determine whether the bacteria containing homologous proteins were Gram‐positive or Gram‐negative.

### Protein structure prediction

The structures of PAAR1, PAAR2, and its cognate immunity proteins were calculated using AlphaFold 3.0^69^. Sequence alignments are generated through CLUSTALW and ESPript 3.0^70^. Models were modified and matched using Chimera X^71^.

## AUTHOR CONTRIBUTIONS

**Hao Wang**: Conceptualization; data curation; formal analysis; investigation; methodology; resources; validation; visualization; writing—original draft. **Ying Liu**: Data curation; methodology; software; visualization. **Zhao Wang**: Conceptualization; data curation; methodology; software; visualization. **PeiYi Xia**: Conceptualization; data curation; methodology; software; visualization. **Zhiwei Li**: Project administration; supervision. **Ming Liu**: Conceptualization; funding acquisition; investigation; methodology; project administration; resources; supervision; writing—original draft; writing—review and editing. **Yang Fu**: Conceptualization; funding acquisition; project administration; supervision; writing—original draft; writing—review and editing.

## ETHICS STATEMENT

This study involved no animal or human experiments. There are no ethical issues involved.

## CONFLICT OF INTERESTS

The authors declare no conflict of interests.

## Supporting information

supmat.

Supplementary Data1.

Supplementary Data 2.

Supplementary Movie 1.

Supplementary Movie 2.

Supplementary Movie 3.

Supplementary Figure 1.

Supplementary Figure 2.

Supplementary Figure 3.

Supplementary Figure 4.

Supplementary Figure 5.

Supplementary Figure 6.

Supplementary Figure 7.

## Data Availability

Data supporting the findings of this study are available within the paper or from the corresponding author upon request. Requests for materials should be addressed to the corresponding authors.

## References

[1] Janda JM , Abbott SL . The genus *Aeromonas*: taxonomy, pathogenicity, and infection. Clin Microbiol Rev. 2010;23:35–73. 20065325 10.1128/CMR.00039-09 PMC2806660

[2] Rasmussen‐Ivey CR , Hossain MJ , Odom SE , Terhune JS , Hemstreet WG , Shoemaker CA , et al. Classification of a hypervirulent *Aeromonas hydrophila* pathotype responsible for epidemic outbreaks in warm‐water fishes. Front Microbiol. 2016;7:1615. 27803692 10.3389/fmicb.2016.01615 PMC5067525

[3] Zhou Y , Yu L , Nan Z , Zhang P , Kan B , Yan D , et al. Taxonomy, virulence genes and antimicrobial resistance of *Aeromonas* isolated from extra‐intestinal and intestinal infections. BMC Infect Dis. 2019;19:158. 30764764 10.1186/s12879-019-3766-0 PMC6376669

[4] Ponnusamy D , Kozlova EV , Sha J , Erova TE , Azar SR , Fitts EC , et al. Cross‐talk among flesh‐eating *Aeromonas hydrophila* strains in mixed infection leading to necrotizing fasciitis. Proc Natl Acad Sci USA. 2016;113:722–727. 26733683 10.1073/pnas.1523817113 PMC4725515

[5] Rasmussen‐Ivey CR , Figueras MJ , McGarey D , Liles MR . Virulence factors of *Aeromonas hydrophila*: in the wake of reclassification. Front Microbiol. 2016;7:1337. 27610107 10.3389/fmicb.2016.01337 PMC4997093

[6] Ma S , Dong Y , Wang N , Liu J , Lu C , Liu Y . Identification of a new effector‐immunity pair of *Aeromonas hydrophila* type VI secretion system. Vet Res. 2020;51:71. 32448355 10.1186/s13567-020-00794-w PMC7245790

[7] Pukatzki S , Ma AT , Sturtevant D , Krastins B , Sarracino D , Nelson WC , et al. Identification of a conserved bacterial protein secretion system in *Vibrio cholerae* using the dictyostelium host model system. ‘Proc Natl Acad Sci USA. 2006;103:1528–1533. 16432199 10.1073/pnas.0510322103 PMC1345711

[8] Ho BT , Dong TG , Mekalanos JJ . A view to a kill: the bacterial type VI secretion system. Cell Host Microbe. 2014;15:9–21. 24332978 10.1016/j.chom.2013.11.008 PMC3936019

[9] Zoued A , Brunet YR , Durand E , Aschtgen MS , Logger L , Douzi B , et al. Architecture and assembly of the Type VI secretion system. Biochim Biophys Acta. 2014;1843:1664–1673. 24681160 10.1016/j.bbamcr.2014.03.018

[10] Costa TRD , Felisberto‐Rodrigues C , Meir A , Prevost MS , Redzej A , Trokter M , et al. Secretion systems in Gram‐negative bacteria: structural and mechanistic insights. Nat Rev Microbiol. 2015;13:343–359. 25978706 10.1038/nrmicro3456

[11] Bönemann G , Pietrosiuk A , Mogk A . Tubules and donuts: a type VI secretion story. Mol Microbiol. 2010;76:815–821. 20444095 10.1111/j.1365-2958.2010.07171.x

[12] Bönemann G , Pietrosiuk A , Diemand A , Zentgraf H , Mogk A . Remodelling of VipA/VipB tubules by ClpV‐mediated threading is crucial for type VI protein secretion. EMBO J. 2009;28:315–325. 19131969 10.1038/emboj.2008.269 PMC2646146

[13] Skaar EP , Wang T , Si M , Song Y , Zhu W , Gao F , et al. Type VI secretion system transports Zn^2+^ to combat multiple stresses and host immunity. PLoS Pathog. 2015;11:e1005020. 26134274 10.1371/journal.ppat.1005020 PMC4489752

[14] Luo Z , Liang X , Pei T‐T , Li H , Zheng H‐Y , Luo H , et al. VgrG‐dependent effectors and chaperones modulate the assembly of the type VI secretion system. PLoS Pathog. 2021;17:e1010116. 34852023 10.1371/journal.ppat.1010116 PMC8668125

[15] Song L , Pan J , Yang Y , Zhang Z , Cui R , Jia S , et al. Contact‐independent killing mediated by a T6SS effector with intrinsic cell‐entry properties. Nat Commun. 2021;12:423. 33462232 10.1038/s41467-020-20726-8 PMC7813860

[16] Liu M , Zhao MY , Wang H , Wang ZH , Wang Z , Liu Y , et al. Pesticin‐like effector VgrG3(cp) targeting peptidoglycan delivered by the Type VI secretion system contributes to *Vibrio cholera*e interbacterial competition. Microbiol Spectr. 2023;11:e0426722. 36625646 10.1128/spectrum.04267-22 PMC9927483

[17] Liu M , Wang H , Liu Y , Tian M , Wang Z , Shu RD , et al. The phospholipase effector Tle1(Vc) promotes *Vbrio cholerae* virulence by killing competitors and impacting gene expression. Gut Microbes. 2023;15:2241204. 37526354 10.1080/19490976.2023.2241204 PMC10395195

[18] Hagan M , Pankov G , Gallegos‐Monterrosa R , Williams DJ , Earl C , Buchanan G , et al. Rhs NADase effectors and their immunity proteins are exchangeable mediators of inter‐bacterial competition in *Serratia* . Nat Commun. 2023;14:6061. 37770429 10.1038/s41467-023-41751-3 PMC10539506

[19] Alcoforado Diniz J , Liu YC , Coulthurst SJ . Molecular weaponry: diverse effectors delivered by the Type VI secretion system. Cell Microbiol. 2015;17:1742–1751. 26432982 10.1111/cmi.12532 PMC4832377

[20] Storey D , McNally A , Åstrand M , Sa‐Pessoa Graca Santos J , Rodriguez‐Escudero I , Elmore B , et al. *Klebsiella pneumoniae* type VI secretion system‐mediated microbial competition is PhoPQ controlled and reactive oxygen species dependent. PLoS Pathog. 2020;16:e1007969. 32191774 10.1371/journal.ppat.1007969 PMC7108748

[21] Wang N , Wu Y , Pang M , Liu J , Lu C , Liu Y . Protective efficacy of recombinant hemolysin co‐regulated protein (Hcp) of *Aeromonas hydrophila* in common carp (*Cyprinus carpio*). Fish Shellfish Immunol. 2015;46:297–304. 26093203 10.1016/j.fsi.2015.06.019

[22] Zhang Y , Huang Y , Ding H , Ma J , Tong X , Zhang Y , et al. A σE‐mediated temperature gauge orchestrates type VI secretion system, biofilm formation and cell invasion in pathogen *Pseudomonas plecoglossicida* . Microbiol Res. 2023;266:127220. 36308833 10.1016/j.micres.2022.127220

[23] Li J , Wu Z , Hou Y , Zhang Y‐A , Zhou Y . Fur functions as an activator of T6SS‐mediated bacterial dominance and virulence in *Aeromonas hydrophila* . Front Microbiol. 2023;13:1099611. 36845974 10.3389/fmicb.2022.1099611 PMC9944043

[24] Cheng AT , Ottemann KM , Yildiz FH . Vibrio cholerae response regulator VxrB controls colonization and regulates the type VI secretion system. PLoS Pathog. 2015;11:e1004933. 26000450 10.1371/journal.ppat.1004933 PMC4441509

[25] Li S , Liu Q , Duan C , Li J , Sun H , Xu L , et al. c‐di‐GMP inhibits the DNA binding activity of H‐NS in *Salmonella* . Nat Commun. 2023;14:7502. 37980414 10.1038/s41467-023-43442-5 PMC10657408

[26] Kitaoka M , Miyata ST , Brooks TM , Unterweger D , Pukatzki S . VasH is a transcriptional regulator of the type VI secretion system functional in endemic and pandemic. J Bacteriol. 2011;193:6471–6482. 21949076 10.1128/JB.05414-11 PMC3232897

[27] Guckes KR , Cecere AG , Williams AL , McNeil AE , Miyashiro T . The bacterial enhancer binding protein VasH promotes expression of a type VI secretion system in *Vibrio fischeri* during symbiosis. J Bacteriol. 2020;202:e00777‐19. 31964698 10.1128/JB.00777-19 PMC7167466

[28] Li J , Wu Z , Wu C , Chen D‐D , Zhou Y , Zhang Y‐A . VasH contributes to virulence of *Aeromonas hydrophila* and is necessary to the T6SS‐mediated bactericidal effect. Front Vet Sci. 2021;8:793458. 34966816 10.3389/fvets.2021.793458 PMC8710571

[29] Mariano G , Trunk K , Williams DJ , Monlezun L , Strahl H , Pitt SJ , et al. A family of type VI secretion system effector proteins that form ion‐selective pores. Nat Commun. 2019;10:5484. 31792213 10.1038/s41467-019-13439-0 PMC6889166

[30] Hespanhol JT , Nóbrega‐Silva L , Bayer‐Santos E . Regulation of type VI secretion systems at the transcriptional, posttranscriptional and posttranslational level. Microbiology. 2023;169:001376. 37552221 10.1099/mic.0.001376 PMC10482370

[31] Manera K , Caro F , Li H , Pei TT , Hersch SJ , Mekalanos JJ , et al. Sensing of intracellular Hcp levels controls T6SS expression In *Vibrio cholerae* . Proc Natl Acad Sci USA. 2021;118:e2104813118. 34161288 10.1073/pnas.2104813118 PMC8237632

[32] Jiang X , Li H , Ma J , Li H , Ma X , Tang Y , et al. Role of type VI secretion system in pathogenic remodeling of host gut microbiota during *Aeromonas veronii* infection. ISME J. 2024;18:wrae053. 38531781 10.1093/ismejo/wrae053 PMC11014884

[33] Pei T‐T , Li H , Liang X , Wang Z‐H , Liu G , Wu L‐L , et al. Intramolecular chaperone‐mediated secretion of an Rhs effector toxin by a type VI secretion system. Nat Commun. 2020;11:1865. 32313027 10.1038/s41467-020-15774-z PMC7170923

[34] Liang X , Pei TT , Wang ZH , Xiong W , Wu LL , Xu P , et al. Characterization of lysozyme‐like effector TseP reveals the dependence of type VI secretion system (T6SS) secretion on effectors in *Aeromonas dhakensis* strain SSU. Appl Environ Microbiol. 2021;87:e0043521. 33837015 10.1128/AEM.00435-21 PMC8174664

[35] Liang X , Moore R , Wilton M , Wong MJQ , Lam L , Dong TG . Identification of divergent type VI secretion effectors using a conserved chaperone domain. Proc Natl Acad Sci USA. 2015;112:9106–9111. 26150500 10.1073/pnas.1505317112 PMC4517263

[36] Seshadri R , Joseph SW , Chopra AK , Sha J , Shaw J , Graf J , et al. Genome sequence of *Aeromonas hydrophila* ATCC 7966T: jack of all trades. J Bacteriol. 2006;188:8272–8282. 16980456 10.1128/JB.00621-06 PMC1698176

[37] Pang M , Jiang J , Xie X , Wu Y , Dong Y , Kwok AHY , et al. Novel insights into the pathogenicity of epidemic *Aeromonas hydrophila* ST251 clones from comparative genomics. Sci Rep. 2015;5:9833. 26014286 10.1038/srep09833 PMC4444815

[38] Jana B , Fridman CM , Bosis E , Salomon D . A modular effector with a DNase domain and a marker for T6SS substrates. Nat Commun. 2019;10:3595. 31399579 10.1038/s41467-019-11546-6 PMC6688995

[39] Tang L , Dong S , Rasheed N , Wu HW , Zhou N , Li H , et al. *Vibrio parahaemolyticus* prey targeting requires autoproteolysis‐triggered dimerization of the type VI secretion system effector RhsP. Cell Rep. 2022;41:111732. 36476863 10.1016/j.celrep.2022.111732

[40] Baslé A , Hewitt L , Koh A , Lamb HK , Thompson P , Burgess JG , et al. Crystal structure of NucB, a biofilm‐degrading endonuclease. Nucleic Acids Res. 2018;46:473–484. 29165717 10.1093/nar/gkx1170 PMC5758888

[41] Potter SC , Luciani A , Eddy SR , Park Y , Lopez R , Finn RD . HMMER web server: 2018 update. Nucleic Acids Res. 2018;46:W200–W204. 29905871 10.1093/nar/gky448 PMC6030962

[42] Ren A , Jia M , Liu J , Zhou T , Wu L , Dong T , et al. Acquisition of T6SS effector TseL contributes to the emerging of novel epidemic strains of *Pseudomonas aeruginosa* . Microbiol Spectr. 2023;11:e0330822. 36546869 10.1128/spectrum.03308-22 PMC9927574

[43] Edwards RA , Keller LH , Schifferli DM . Improved allelic exchange vectors and their use to analyze 987P fimbria gene expression. Gene. 1998;207:149–157. 9511756 10.1016/s0378-1119(97)00619-7

[44] Santin YG , Doan T , Journet L , Cascales E . Cell width dictates type VI secretion tail length. Curr Biol. 2019;29:3707–3713.e3. 31630952 10.1016/j.cub.2019.08.058

[45] Vettiger A , Basler M . Type VI secretion system substrates are transferred and reused among sister cells. Cell. 2016;167:99–110.e12. 27616061 10.1016/j.cell.2016.08.023

[46] Liang X , Zheng HY , Zhao YJ , Zhang YQ , Pei TT , Cui Y , et al. VgrG spike dictates PAAR requirement for the assembly of the type VI secretion system. J Bacteriol. 2023;205:e0035622. 36655996 10.1128/jb.00356-22 PMC9945574

[47] Pissaridou P , Allsopp LP , Wettstadt S , Howard SA , Mavridou DAI , Filloux A . The *Pseudomonas aeruginosa* T6SS‐VgrG1b spike is topped by a PAAR protein eliciting DNA damage to bacterial competitors. Proc Natl Acad Sci USA. 2018;115:12519–12524. 30455305 10.1073/pnas.1814181115 PMC6298103

[48] Wang N , Liu J , Pang M , Wu Y , Awan F , Liles MR , et al. Diverse roles of Hcp family proteins in the environmental fitness and pathogenicity of *Aeromonas hydrophila* Chinese epidemic strain NJ‐35. Appl Microbiol Biotechnol. 2018;102:7083–7095. 29862449 10.1007/s00253-018-9116-0

[49] Kennedy NW , Comstock LE . Mechanisms of bacterial immunity, protection, and survival during interbacterial warfare. Cell Host Microbe. 2024;32:794–803. 38870897 10.1016/j.chom.2024.05.006 PMC11216714

[50] Sun Y , Wang L , Zhang M , Jie J , Guan Q , Fu J , et al. *Acinetobacter nosocomialis* utilizes a unique type VI secretion system to promote its survival in niches with prey bacteria. mBio. 2024;15:e0146824. 38916378 10.1128/mbio.01468-24 PMC11253628

[51] Froquet R , Lelong E , Marchetti A , Cosson P . *Dictyostelium discoideum*: a model host to measure bacterial virulence. Nat Protoc. 2008;4:25–30. 10.1038/nprot.2008.212 19131953

[52] Jiang K , Li W , Tong M , Xu J , Chen Z , Yang Y , et al. *Bacteroides fragilis* ubiquitin homologue drives intraspecies bacterial competition in the gut microbiome. Nat Microbiol. 2024 Jan;9:70–84. 38082149 10.1038/s41564-023-01541-5

[53] Ma J , Sun M , Dong W , Pan Z , Lu C , Yao H . PAAR‐Rhs proteins harbor various C‐terminal toxins to diversify the antibacterial pathways of type VI secretion systems. Environ Microbiol. 2017;19:345–360. 27871130 10.1111/1462-2920.13621

[54] Jurėnas D , Rey M , Byrne D , Chamot‐Rooke J , Terradot L , Cascales E . *Salmonella* antibacterial Rhs polymorphic toxin inhibits translation through ADP‐ribosylation of EF‐Tu P‐loop. Nucleic Acids Res. 2022;50:13114–13127. 36484105 10.1093/nar/gkac1162 PMC9825190

[55] González‐Magaña A , Tascón I , Altuna‐Alvarez J , Queralt‐Martín M , Colautti J , Velázquez C , et al. Structural and functional insights into the delivery of a bacterial Rhs pore‐forming toxin to the membrane. Nat Commun. 2023;14:7808. 38016939 10.1038/s41467-023-43585-5 PMC10684867

[56] Dong TG , Ho BT , Yoder‐Himes DR , Mekalanos JJ . Identification of T6SS‐dependent effector and immunity proteins by Tn‐seq in *Vibrio cholerae* . Proc Natl Acad Sci USA. 2013;110:2623–2628. 23362380 10.1073/pnas.1222783110 PMC3574944

[57] Zhu L , Xu L , Wang C , Li C , Li M , Liu Q , et al. T6SS translocates a micropeptide to suppress STING‐mediated innate immunity by sequestering manganese. Proc Natl Acad Sci USA. 2021;118:e2103526118. 34625471 10.1073/pnas.2103526118 PMC8545469

[58] Luo J , Chu X , Jie J , Sun Y , Guan Q , Li D , et al. *Acinetobacter baumannii* kills fungi via a type VI DNase effector. mBio. 2023;14:e0342022. 36625573 10.1128/mbio.03420-22 PMC9973263

[59] Khan SR , Gaines J , Roop RM , Farrand SK . Broad‐host‐range expression vectors with tightly regulated promoters and their use to examine the influence of TraR and TraM expression on Ti plasmid quorum sensing. Appl Environ Microbiol. 2008;74:5053–5062. 18606801 10.1128/AEM.01098-08 PMC2519271

[60] Schindelin J , Arganda‐Carreras I , Frise E , Kaynig V , Longair M , Pietzsch T , et al. Fiji: an open‐source platform for biological‐image analysis. Nat Methods. 2012;9:676–682. 22743772 10.1038/nmeth.2019 PMC3855844

[61] Sullivan MJ , Petty NK , Beatson SA . Easyfig: a genome comparison visualizer. Bioinformatics. 2011;27:1009–1010. 21278367 10.1093/bioinformatics/btr039 PMC3065679

[62] Wang J , Chitsaz F , Derbyshire MK , Gonzales NR , Gwadz M , Lu S , et al. The conserved domain database in 2023. Nucleic Acids Res. 2023;51:D384–D388. 36477806 10.1093/nar/gkac1096 PMC9825596

[63] Fey P , Kowal AS , Gaudet P , Pilcher KE , Chisholm RL . Protocols for growth and development of *Dictyostelium discoideum* . Nat Protoc. 2007;2:1307–1316. 17545967 10.1038/nprot.2007.178

[64] Kumar S , Stecher G , Li M , Knyaz C , Tamura K . MEGA X: molecular evolutionary genetics analysis across computing platforms. Mol Biol Evol. 2018;35:1547–1549. 29722887 10.1093/molbev/msy096 PMC5967553

[65] Xie J , Chen Y , Cai G , Cai R , Hu Z , Wang H . Tree visualization by one table (tvBOT): a web application for visualizing, modifying and annotating phylogenetic trees. Nucleic Acids Res. 2023;51:W587–W592. 37144476 10.1093/nar/gkad359 PMC10320113

[66] Katoh K , Standley DM . MAFFT multiple sequence alignment software version 7: improvements in performance and usability. Mol Biol Evol. 2013;30:772–780. 23329690 10.1093/molbev/mst010 PMC3603318

[67] Nguyen LT , Schmidt HA , von Haeseler A , Minh BQ . IQ‐TREE: a fast and effective stochastic algorithm for estimating maximum‐likelihood phylogenies. Mol Biol Evol. 2015;32:268–274. 25371430 10.1093/molbev/msu300 PMC4271533

[68] Söhngen C , Podstawka A , Bunk B , Gleim D , Vetcininova A , Reimer LC , et al. BacDive—the bacterial diversity metadatabase in 2016. Nucleic Acids Res. 2016;44:D581–D585. 26424852 10.1093/nar/gkv983 PMC4702946

[69] Abramson J , Adler J , Dunger J , Evans R , Green T , Pritzel A , et al. Accurate structure prediction of biomolecular interactions with AlphaFold 3. Nature. 2024;630:493–500. 38718835 10.1038/s41586-024-07487-w PMC11168924

[70] Robert X , Gouet P . Deciphering key features in protein structures with the new ENDscript server. Nucleic Acids Res. 2014;42:W320–W324. 24753421 10.1093/nar/gku316 PMC4086106

[71] Mirdita M , Schütze K , Moriwaki Y , Heo L , Ovchinnikov S , Steinegger M . ColabFold: making protein folding accessible to all. Nat Methods. 2022;19:679–682. 35637307 10.1038/s41592-022-01488-1 PMC9184281

