# Supplementary material for: Mechanistic insights into the T6SS of multi‐drug‐resistant Aeromonas hydrophila and its role in competition and pathogenesis
Source: mLife. 2025 Jul 22;4(4):363–77. doi: 10.1002/mlf2.70018 (PMC12396204; doi:10.1002/mlf2.70018)
Supplement: Supplementary file 1 — supmat. [file MLF2-4-363-s004.docx]

**Supplementary Figure 1 PCR verification of specific T6SS effectors in *A. hydrophila* clinical isolates.** Gel map of PCR amplifying products from ATCC 7699, AH17, AH18 and AH54 using specific effector primers. The amplifying products were confirmed by sequencing.

**Supplementary Figure 2 Phylogenetic analyses of PAAR1 and PAAR2 with representative members of the families Tle1-4.** The evolutionary history was inferred using the Maximum Likelihood method, and the bootstrap replications were 1000. Protein sequences are provided in file 1 in the supplemental data. The figure was processed using MEGA-X and iTOL.

**Supplementary Figure 3 Growth curve assay of AH54 WT and Δ*vasK*.** Overnight cultures of AH54 and Δ*vasK* were inoculated into the fresh LB medium, grown at 28℃. The OD_600_ was monitored at time points indicated.

**Supplementary Figure 4 Low temperature promotes upregulation of T6SS-related gene expression in AH54.** **(A to H)** The overnight cultured bacteria were transfected according to the ratio of 1:100, and placed in 22 °C, 28 °C, 37 °C for culture, respectively, and wait for the growth OD_600_ to 1.5, collect 4 mL of bacteria, and carry out the TRIZOL method to extract the RNA, and reverse transcription to get the cDNA. The qPCR was subsequently conducted. The WT at 37℃ was used as the standard to homogenise the other groups, and the endogenous reference gene was 16sRNA.

**Supplementary Figure 5 AH54 has T6SS secretory activity at 37 °C.** AH54 was grown in LB medium to an OD_600_ of 1.5 at 37℃. Collect cell pellets and 20 mL supernatants were analyzed by SDS-PAGE and immunoblot assays using anti-Hcp and anti-RpoB primary antibodies.

**Supplementary Figure 6 Sequence and interaction alignment of PAAR1 with PAAR2 and Tsi3 with Tsi4. (A and B)** The protein sequence alignment was performed using ClustalX. **(C)** Interaction predictions were made by AlphaFold 3.0 for RAAR1 and Tsi3, and PAAR2 and Tsi4, and structural analyses were performed using Chimera X. **(D)** Using the Chimera X default parameters shows the amino acid residues involved in the interaction.

**Supplementary Figure 7 Rhs2 effector protein undergoes self-cleavage. (A and B)** *E. coli* BL21(DE3) cells harboring p*rhs2* and p*rhs2-CT* after IPTG induction were subjected to protein purification and SDS-PAGE. T: Supernatant; FT: Filtration of the supernatant; W: Washing; E: Elution.

**Supplementary Movie 1 Measurement of T6SS dynamic in *A. hydrophila* AH54.** Sheath assembly of AH54 *vipA*_sfGFP was visualized by green fluorescent channel. Phase and GFP images were acquired at 30-second intervals. Data is related to Figure 2A.

**Supplementary Movie 2 and 3 Time-lapse confocal microscope assay between predator *A. hydrophila* AH54 and prey *E. coli* MG1655.** MG1655 was co-incubated with either AH54 *vipA*_sfGFP WT (Movie S2) or AH54 *vipA*_sfGFP Δ*vasK* (Movie S3) on a 1% LB agarose pad. Phase, PI, and GFP images were acquired at 2-min intervals (overlays depicted in movie sequence). The AH54 strain appears in the green channel and PI staining in the red channel. Data is related to Figure 2E.
