# Supplementary material for: Mechanistic insights into the T6SS of multi‐drug‐resistant Aeromonas hydrophila and its role in competition and pathogenesis
Source: mLife. 2025 Jul 22;4(4):363–77. doi: 10.1002/mlf2.70018 (PMC12396204; doi:10.1002/mlf2.70018)
Supplement: Supplementary file 2 — Supplementary Data1. [file MLF2-4-363-s007.docx]

Supplementary Information

Table S1 Antibiotic susceptibilities of *Aeromonas* *hydrophila* strains used in this study[1].

| Antimicrobial category | Antimicrobial agent | AH17 | AH18 | AH54 |
| --- | --- | --- | --- | --- |
| Fluoroquinolones | ciprofloxacin | >4 (R) | >4 (R) | >4 (R) |
|  | nalidixic acid^b^ | >32 (R) | >32 (R) | >32 (R) |
| β-lactamase | ampicillin^b^ | >32 (R) | >32 (R) | >32 (R) |
|  | amoxicillin-clavu | >32 (R) | >32 (R) | >32 (R) |
|  | ceftriaxone | >8 (R) | >8 (R) | >8 (R) |
|  | cefoxitin | >32 (R) | >32 (R) | >32 (R) |
|  | cefepime | >32 (R) | 2 (S) | 2 (S) |
|  | imipenem | 1 (S) | 1 (S) | 1 (S) |
| Amphenicols | chloramphenicol | <2 (S) | 4 (S) | <2 (S) |
| Sulfanilamide | sulfonamides^b^ | >512 (R) | >512 (R) | 256 (S) |
|  | sulfamethoxazole | 0.25 (S) | 0.25 (S) | 0.25 (S) |
| Tetracyclines | tetracycline | <2 (S) | <2 (S) | <2 (S) |
|  | doxycycline^b^ | 2 (S) | 2 (S) | 2 (S) |
| Aminoglycosides | gentamicin | >32 (R) | >32 (R) | 2 (S) |
| Macrolides | azithromycin^b^ | >64 (R) | >64 (R) | >64 (R) |

^a^ Breakpoints are based on the CLSIM45-A3 standards for *Aeromonas* spp.

^b^ Other breakpoints refer to the CLSIM100- ED33 criteria for *Enterobacteriaceae*.

Table S2. Strains and plasmids used in this study

| Species | Genotype/strain | Description | | Source |
| --- | --- | --- | --- | --- |
| *A*. *hydrophila* | ATCC 7966 | Reference strain and serving as prey in competition assay | | Lab stock |
|  | AH54 | Clinical isolation of multi-drugs resistant strain | | [1] |
|  | AH17 | Clinical isolation of multi-drugs resistant strain | | [1] |
|  | AH18 | Clinical isolation of multi-drugs resistant strain | | [1] |
|  | Δ*vasK* | T6SS inactivation, in-frame deletion of the *vasK* | | This study |
|  | Δ*rhs1* | In-frame deletion of the *rhs1* | | This study |
|  | Δ*rhs2* | In-frame deletion of the *rhs2* | | This study |
|  | Δ*rhs1/*Δ*rhs2* | In-frame deletion of the *rhs1* and *rhs2* | | This study |
|  | Δ*PAAR1* | In-frame deletion of the *PAAR1* | | This study |
|  | Δ*PAAR2* | In-frame deletion of the *PAAR2* | | This study |
|  | Δ*PAAR1/*Δ*PAAR2* | In-frame deletion of the *PAAR1* and *PAAR2* | | This study |
|  | Δ*rhs1/tsi1* | In-frame deletion of the *rhs1* and *tsi1* | | This study |
|  | Δ*rhs2/tsi2* | In-frame deletion of the *rhs2* and *tsi2* | | This study |
|  | Δ*PAAR1/tsi3* | In-frame deletion of the *PAAR1* and *tsi3* | | This study |
|  | Δ*PAAR2/tsi4* | In-frame deletion of the *PAAR2* and *tsi4* | | This study |
|  | Δ*PAAR1/tsi3/ PAAR2/tsi4* | In-frame deletion of the *PAAR1*, *tsi3*, *PAAR2* and *tsi4* | | This study |
|  | *vipA_sfGFP* | Fusion of the *sfGFP* coding sequence at the 3' of *vipA* in AH54 | | This study |
| *E*. *coli* | SM10*λpir* | For conjunction assays | | Lab stock |
|  | MG1655 | Serving as prey in competition assay | | Lab stock |
|  | BL21(DE3) | Host bacteria of protein expression and purification | | Lab stock |
|  | DH5*α* | Construction of recombination plasmid | | Lab stock |
| *V.vulnificus* | Vv55 | Serving as prey in competition assay | | Lab stock |
| *V. parahaemolyticus* | Vp1360 | Serving as prey in competition assay | | Lab stock |
| *D. discoideum* |  | Serving in the phagocytosis *Dictyostelium discoideum* model | | Lab stock |
|  |  | |  | |
| Plasmid |  | |  | |
| pRE112 | Suicidal conjugation vector for chromosomal allelic changes Cm^R^ | | [2] | |
| pSRKTc | pBBR1MCS-3-derived broad-host-range expression vector containing *lac* promoter and *lacI^q^*, *lacZα+*, and Tet^R^ | | [3] | |
| pUC19 | Standard *E. coli* vector with a multiple cloning site (MCS) for DNA cloning | | Lab stock | |
| pET28a | Expression vector with N-terminal hexahistidine affinity tag, Km^R^ | | Lab stock | |
| pRE112-Δ*vasK* | Recombinant vector to construct chromosomal clean deletion of *vasK* | | This study | |
| pRE112-Δ*rhs1* | Recombinant vector to construct chromosomal clean deletion of *rhs1* | | This study | |
| pRE112-Δ*rhs2* | Recombinant vector to construct chromosomal clean deletion of *rhs2* | | This study | |
| pRE112-Δ*PAAR1* | Recombinant vector to construct chromosomal clean deletion of *PAAR1* | | This study | |
| pRE112-Δ*PAAR2* | Recombinant vector to construct chromosomal clean deletion of *PAAR2* | | This study | |
| pRE112-*vipA_sfGFP* | Recombinant vector to insert the *sfGFP* coding sequence at the 3' of *vipA* | | This study | |
| pSRKTc-*tsi1* | For expressing immunity protein Tsi1, IPTG inducible | | This study | |
| pSRKTc-*tsi2* | For expressing immunity protein Tsi2, IPTG inducible | | This study | |
| pSRKTc-*tsi3* | For expressing immunity protein Tsi3, IPTG inducible | | This study | |
| pSRKTc-*tsi4* | For expressing immunity protein Tsi4, IPTG inducible | | This study | |
| pSRKTc-*PAAR1* | For expressing effector protein PAAR1, IPTG inducible | | This study | |
| pSRKTc-*PAAR2* | For expressing effector protein PAAR2, IPTG inducible | | This study | |
| pET28a-*rhs2* | For purifying effector protein Rhs2, IPTG inducible | | This study | |
| pET28a-*rhs2-CT* | For purifying effector protein C-terminal Rhs2-CT, IPTG inducible | | This study | |

Table S3. Primers used in this study.

| Primer name | Sequence |
| --- | --- |
| pRE112-Δ*vasK*- A | CTCGATATCGCATGCGGTACCGGTGGCCCAGAAGCTCGG |
| pRE112-Δ*vasK*- B | CCCGACGCGAAAAATGTGATCCAACTCCGACTCAATAAC |
| pRE112-Δ*vasK*- C | ATCACATTTTTCGCGTCGGGGGC |
| pRE112-Δ*vasK*- D | GGCCCGATCCCAAGCTTCTTCTAGATGTGCAGCTTCTCGCTGATG |
| pRE112-Δ*rhs1*- A | CTCGATATCGCATGCGGTACCCCGGTCGGATTGGCCC |
| pRE112-Δ*rhs1*- B | ATCGATATCTCGATATTGTTGCGCTCATCGCGCAC |
| pRE112-Δ*rhs1*- C | GCAACAATATCGAGATATCGATTATACCCAATGCACAACACA |
| pRE112-Δ*rhs1*- D | GGCCCGATCCCAAGCTTCTTCTAGAGTCAGAATTTTATATATACCTCATGAAAATCAAAGTGTTCTGG |
| pRE112-Δ*rhs2*- A | CTCGATATCGCATGCGGTACCGTCAATCATCTATTTTACCTGGGTAGACGG |
| pRE112-Δ*rhs2*- B | ACAATATCCTGATAATGGGCATCCATACATCGTGACA |
| pRE112-Δ*rhs2*- C | GCCCATTATCAGGATATTGTTGCGCTCATCGC |
| pRE112-Δ*rhs2*- D | GGCCCGATCCCAAGCTTCTTCTAGACCGGTCGGATTGGCCC |
| pRE112-Δ*PAAR1*- A | CTCGATATCGCATGCGGTACCCAAGGTTGGGCGGGTGGC |
| pRE112-Δ*PAAR1*- B | TGGTAATGGTGCCACCGGCACAGCTATCACCTA |
| pRE112-Δ*PAAR1*- C | TAGCTGTGCCGGTGGCACCATTACCAGCAAACT |
| pRE112-Δ*PAAR1*- D | GGCCCGATCCCAAGCTTCTTCTAGAGTACTTCGGCAGGCGTCAAC |
| pRE112-Δ*PAAR2*- A | CTCGATATCGCATGCGGTACCACAGACTATCTTCTACCGAGAAAGCTAAAT |
| pRE112-Δ*PAAR2*- B | AGGAGTCATTTGCAGCTGGCAAGATGTTACC |
| pRE112-Δ*PAAR2*- C | GCCAGCTGCAAATGACTCCTTAACGACGATCCG |
| pRE112-Δ*PAAR2*- D | GGGCCCGATCCCAAGCTTCTTCTAGAATGATGGCGGTGCCGCGTATC |
| pRE112-*vipA_sfGFP*- A | CTCGATATCGCATGCGGTACCATGGGAAAAGGAATTGACGGCG |
| pRE112-*vipA_sfGFP*- B | CTCCTGCGGCCGCCTCGGCAGGCTTAATCAA |
| pRE112-*vipA_sfGFP*- C | CTGCCGAGGCGGCCGCAGGAGGAGGATC |
| pRE112-*vipA_sfGFP*- D | CCAACCCGGCGATTACTTGTACAGCT |
| pRE112-*vipA_sfGFP*- E | TACAAGTAATCGCCGGGTTGGATTGACTGA |
| pRE112-*vipA_sfGFP*- F | GGCCCGATCCCAAGCTTCTTCTAGACTGGCCACCGAACTGACC |
| pSRKTc-*tsi1*- A | ATTTCACACAGGAAACAGCATATGATGAAATTTTACTTAATGAATAATGTCAAGACCCC |
| pSRKTc-*tsi1*- B | GCCCGGGGGATCCACTAGTTCTAGATCAATCATCTATTTTACCTGGGTAGACGGAG |
| pSRKTc-*tsi2*- A | ATTTCACACAGGAAACAGCATATGATGGCACTAAAGTACTCTTTAGGATTAAGTG |
| pSRKTc-*tsi2*- B | GCCCGGGGGATCCACTAGTTCTAGATCAATCATCTATTTTACCTGGGTAGACGG |
| pSRKTc-*tsi3*- A | GCCCGGGGGATCCACTAGTTCTAGAGTTAGACCTTCTCACCCTTGG |
| pSRKTc-*tsi3*- B | ATTTCACACAGGAAACAGCATATGAATGATATGGGTTTCTCGTTTAAAAGTACTTTGC |
| pSRKTc-*tsi4*- A | GCCCGGGGGATCCACTAGTTCTAGACTATATCTTCTCTCCTCGGGCC |
| pSRKTc-*tsi4*- B | ATTTCACACAGGAAACAGCATATGATGATATGGTTAGCTCGTTTGAAGGTG |
| pSRKTc-*PAAR1*-A | ATTTCACACAGGAAACAGCATATGATGCCAAGTGCTGCCC |
| pSRKTc-*PAAR1*-B | GCCCGGGGGATCCACTAGTTCTAGATCATTTTACTCCTCCAAGTTTGCTG |
| pSRKTc-*PAAR2*-A | ATTTCACACAGGAAACAGCATATGATGCCAAGTGCTGCCC |
| pSRKTc-*PAAR2*-B | GCCCGGGGGATCCACTAGTTCTAGATCATTTTACTCCTCCCAGCTTACT |
| pET28a-*rhs2*-A | TGGACAGCAAATGGGTCGCGGATCCATGAGTAAAAATAAAACAGCAAGTCTGATGT |
| pET28a-*rhs2*-B | AGTGGTGGTGGTGGTGGTGCTCGAGTCACTTAATCCTAAAGAGTACTTTAGTGCCATCA |
| pET28a-*rhs2-CT*-A | TGGACAGCAAATGGGTCGCGGATCCCCGTTAGAGTGGGTAGATCCGC |
| pET28a-*rhs2-CT*-B | AGTGGTGGTGGTGGTGGTGCTCGAGTCACTTAATCCTAAAGAGTACTTTAGTGCCATCA |
| *rhs1*-A | TGGACAGCAAATGGGTCGCGGATCCCCGCTGGAGTGGGTAGATCC |
| *rhs1*-B | AGTGGTGGTGGTGGTGGTGCTCGAGTCATCTCTTACCTCCACAGGACTTATATCTTTTT |
| *rhs2*-A | TGGACAGCAAATGGGTCGCGGATCCCCGTTAGAGTGGGTAGATCCGC |
| *rhs2*-B | AGTGGTGGTGGTGGTGGTGCTCGAGTCACTTAATCCTAAAGAGTACTTTAGTGCCATCA |
| *PAAR1*-A | ATGCCAAGTGCTGCCCGATTAGGT |
| *PAAR1*-B | ACGCAAAGTACTTTTAAACGAGAAACCCATA |
| *PAAR2*-A | ATGCCAAGTGCTGCCCGATTAGGT |
| *PAAR2*-B | CAGAGCACCTTCAAACGAGCTAACCATA |
| Q-*16srRNA*-AH-R | CGGTAATACGGAGGGTGCAA |
| Q-*16SrRNA*-AH-F | CTGGAATTCTACCCCCCTCTACAA |
| Q-*hcp1*-AH-F | CATTTTCGTGCAGGGTCACGAA |
| Q-*hcp1*-AH-R | TGGACACGCTGACCAGCA |
| Q-*hcp2*-AH-F | CACCGTGGCCCTGAACAA |
| Q-*hcp2*-AH-R | GGCATCAGTCAGCACGGT |
| Q-*hcp3*-AH-F | GCCAGTGCGTTGTACATCA |
| Q-*hcp3*-AH-R | CGAGATGCTGGTGCAAGAGT |
| Q-*vipA*-AH-F | CCGATCGAGGAGCGTCAGA |
| Q-*vipA*-AH-R | GGATCTTGATCGAAACCGGCA |
| Q-*PAAR1/2*-AH-F | CTCCATGCTTGCCCTTGT |
| Q-*PAAR1/2*-AH-R | CATCCCCCACCCTTGCT |
| Q-*rhs1/2*-AH-F | CCGAATTTATGATATGGGCTGCGA |
| Q-*rhs1/2*-AH-R | AAGTATTCACCAGCCAACGCA |
| Q-*vipB*-AH-F | CCAGGTGCTGCATGCCA |
| Q-*vipB*-AH-R | GCCACCGAACTGACCGT |
| Q-*vasH*-AH-F | CACGGTTGGTGCACCAGT |
| Q-*vasH*-AH-R | GCCCGGTCTTGTTGGCA |

File 1. Representative sequences of PAAR1 and PAAR2 homologs

>AKJ35788.1

MAISAHCIPCEKNNCWIEIDVRDEQNRSFKGQKATLTDATGTSKTVTLKDGPTLVQGFAVGPVTVKLETQPWLKVAQSREALKEGETSQVPAYTDKLFGHDDVKREHIKVTSGDLCLTDPEQPLPEGHKAGQAQPPRFITKHSYVIEVKGYQLTTLRIGVFFDGTANNTFKHREGKSALETALAQCSPEEQQVLLEQCFEGALPDGLNNSEKNDITNIGKTHELYKPPTKTELNVAVYIEGIGTTQGEGDTSMGLGADKGETSSASRVEEACRTQIVAEVKKRLERILPTIECIHKVEFDVFGFSRGASAARQFVNRIDKKGDHPLVESMAADPDIPLKAGFDWASRDDVRIQFVGLFDTVVSSYLWGKRNVALAPDCAERVVHIVAADEWRYHFDLTRITDDAAGTALAENFTEVIIPGAHSDIGGGYYSRWSLRDPNYASPLITENSVIATFSSNELPHIHPADAQAYRDASAYAKMRAGQGWGKGVVSLASHSDATRLGYLNVRPRSLGPITGSQSGGLRNISVDVVLHRVVEGEYSRIPLHMMVEAARDAGVPFKKWVSQGDNAYRLDSALSYPTVNLAKLDELWVDVAQRRGEVINLARSLPDEVYQKLRLSYLHYSADTGLVNKPNRVGDKEIRKLTGNQKGGY

>YP_001478026.1

MSEINTDLAWLPPAFPAQGRLPTQAALVGANCAQQDSHELAYRQALCLAAGRRVEPPCCKTLHVSLFFDGTGNNLNHDLYIADPKHPTNIARLFRATIGQGAAGGVSKGPELLDADGSGEDKYYKYYIPGVGTPFPEVNDLDFTMMGLAVATHGEDRINWGLLRLLDALKRTMTRKSLSDDESWKAVDKMATSMASFGLTGSANRFETFQRLLKDMSPDLQKALMPAEPGKPKLLGIKLYVYGFSRGAAAARAFVRWLSELLPKPEEGQDRPEQYLAVGGLKIPLSVEFLGLLDTVASVGVAHVAPVAEGHMGWADGTQELPAEKIYGGLIKKCVHLVSSHEQRLCFPLDSIRRPDGTYPANSQEVVYPGMHSDLGGGYPPGDQGKANDEFDRFLLSQLALHDLYASAFNSGAPLKVSPPSLPTDLQKDIWRQMSPELQLEFAVAPELINRFNAWRELTLGLTVPLQPLSPEQAAKYDPPRAPVSLEKAVENQLGWITAWRINRYAGGSYKTQRFYVDSAANGLDKDSDPLVRKQSEAARKALQKEVDDVRRDMKAQHNPEEGFLKLPPGPKDFDAALGQTQLRQAAEEFREDYHGLSRTSTGNWLFTVADSVNNAIFLLNNDDEYGEWLRIKTAGDDRVKVLFPISGDASSATQSAGLVRALFDDQIHDSRAWFMHNAFGSREPWGSYFLYRMIYFGSRSSKPMTPLMIAGAVVGVATLAAGVAVIIKQKSAKGKLAGLVGTAGAIYLETQAVDLLSGKPLPMLPNAAQLQAPTMEPGVVVAQQTQAVAEQRLALAKSLIESGWAERLKSTVTA

>ZP_05133435.1

MPANGQRALSGAEAQQRARAMACLREKGSECQGQVHVSIYFDGTGNNREWEGTFVTGKTRSPKTQLARNGHSNVARLYDAALKERENGFFSIYVPGVGTPFADVGDTNQDGDTLGGGAARYGADRIHWAILQIINSVHQYLNASDLIQPNEMKVLVASMSETRLLEGMARRSMLTAIAQRLERVVNGHQRRVKSVHVSVFGFSRGAAQARAFVHRLYETAEAWGSGCGYNIAGIPLYLNFMGIFDTVASVGVAAMSRVSKGKWDWAAGDMMSIHPEARQCVHFAALHEQRINFPLDLATSGREVLYPGMHSDVGGGYSPGGQGKDFVSGSADGKAKLSQIPLIDMHHEAVKAGVIVKTIDEIGTRPTLAMHFGCHPQLIRDYNAWLTGHGVPGGGHAQQIAGHCRQYVAWKGKRLPNGPQSVLQQPFFTQSDAEDQVDLANAQRDFANLVGKLSRGKQEMAAYRSQLDESQKRMEAGRKAGRPVFEPTPRASQAAYDYAGIPAETSTLLNLVLDGAPVPDVSTNLFDNYMHDSLAGFYIGKWTELNIPAVSTYGYLRYREVFSVAGRRAQECRDPATLPPANIPSIGGAFQQLGTAMGG

>YP_002396321.1

MSSKISGLAFPCYAPPVFPEDGRLILSEAQVNANYLKQINKTEEHKTNCSKQAGFRIGFTCNQSLHISLFFDGTNNNEYNDTPGHPTNIAKLFHTTYQNAEEQGYFNYYIPGVGTPFPKIGEMDYSNSGLEFATGGEDRINWALLRLVDALSYSIDPSHKRLDDNVAKEHIPLMRAPWPMTGEVNRRNVINPYLEKLQGLLEQASPRLLNVKLFIYGFSRGAAEARTFVNWLTQLACPQEQQVMLAGLAVSIEFLGLLDTVASVGAAHVLPGAAGHMGWADSTQQLPDERQFPGLIKCCRHFVAAHEQRLCFPLDSIRRPDGNYPLNAEEIIYPGMHSDVGGGYPVRDQGKSCGDSGDILSQIALHDMYLAAFDSGAPLAVYSKFVTPLIKGVSPLRIMSPSSVKEFTIANSLTKRFNIWRQTLLNTTLQGTEEMIDTREGYHPYQLAQVLQQIKAMGDGLYPQFFIDSLGTGTRTQPCAELLALYDDQVHDSRAWFMQSSLGGREPWGGYFRYRMIYFGDEANKELKLISADGEVVGDQPTSNRVIYWMESKTVSRG

>NP_251980.1

MPNFGFHIAPTHPVAGRLTYDSKKLSENILKQQSDERVFSRAQEQKRLSEGDVVGGAPCCKAIHITLGFDGTNNNDKADGSSVSPSCSNVARLIHASIGSGDDINSRGIFKYYCPGVGTVFPDIKEFTPSNMGLIGAEGGENRINWGLVQLVDALFYTLLKSRLKLNDVQGLVEEMSTNWTVSTLTGGLLENGEKKRRAALEPKLKELEEKLRQRQNSGQKPHILAMRLYIYGFSRGAAEARAFANWLQELTRVSDADGRVEYRFAGLPISIEFLGLFDTVAAVGLADSAPFAAGHMDWADDTMRLPDEALSQCLPTILPEDCSFLKRCVHLVSCHEQRASFPLDSIRRRDMDANGRRTGPSCYRKWTVEYAYPGVHSDVGGGYGVGNQGKAVGGSEFLLSQIALQHMYAEAFEAGAPLQVPAPAVHPDFHEEWRVMVPKIEAEFSVSEELATRFNAWQAQAKAGPLEEVIRRETALITAWRIDRYAGGLRNKAFFANVPPDMPEAQQKAWEALHKRRSREYAAAQQGEPLPPMSAAEQAEWDRNVALIGGEDQLRDLRVEKQFDPPLDQRQLLGAAAEFAHDYKGDWGVLDDGMTVGGVIDLLLGGTVFLINEEDEAEEYSQIHRDGSARYHQLFSAPDRVAPGQEKLVALFDEQVHDSRAWFMNTSAIGPREPFTDYFRYRLVHFDNESNKRLSVLATAGRVVGVGVMLASVGLSVKRRDPRMLLGLFLPSLARPLLSGKVGLPEISAFDPLTGIALPMVGGAALDNLRAFTREPGDKVEQIGQLPPPPPLAVAAVQSPALQQVLLAQQTVEALKARDLGSLAGLVAKAELTQAPAAATPAWLAEAKQALQDMGTEQAQPPGPGSAPGWLKRGKDLMESL

>ZP_05828070.1

MTQAIQNETRSVRDMLIAAKVLAKDGKATESPCKTCRVPVWVSFFFDGTGNNKDADAATLNQSNVVALFEAHKQDSKNGIEKFYYEGLGTQFRFDKYSVVDSGKITAAARSLQGRKIDITDAEWRKQGYSESGKGVQGALGLGVALGIKQRLQKAIFELVDYLDKIYTQKGITEINISAFGFSRGATEARIFMNWLQHAPNVTTQGTGSGKKLFYRGKPLKAKFLGIFDTVESIGNAAQNKNPELYRTRIEDYIEHSMHLVASLEMRQSFPLTPTGKPTANTVKGLIHDQKVYPGVHSNVGGGYMPMEQARILGLSRITLHAMYNRACAYGLKFFTLNELNAAKQRKIVFTRFYAFDSKWQQDLNNFMAYVKGGTSFEQQMQGQIALYHQWIREGGYARFIHRKTRERIGRKEKITAITKLNDGLFENIRQALNVYVPEGARPYDVIKGRDRKSTLPKEVIYYFENYVCDSVGGFIAEASDFQAILNDGKAPNYFIPRGIVRPT

>YP_443213.1

MNFRFAPAERPDIGVVTKEEKDAILRRLHDDDGMSCCKTLHIGIFFDGTRNNAERDKSGHKHSNVARLRDAFPQDRYHKSIYVAGVGTPFSSEIGDYGIGLQAVAGASAGWAGEGRINWALLQIHNAVHECAFRVGLSTALGVDDKNLVKLMSLDMNFKGIDLGGNAPQPGSTGDIKSRSSPGIGALKLIAAEQYGAELTWDKDTNWSQLKDDLDSSKWAAAVRAWDGRRRKILGDRRAQLKARVGDMLVKGKPRIQRIRLYVFGFSRGAAEARTFSNWLVDALESDFSLCGVPVSYDFLGIFDTVASVGIAQSAAATLFDGHGGWARKELMAVPHYVRRCVHMVAAHEPRGSFPLDLIDCSLEGREEIVYPGVHSDVGGGYGPAEQGRGRGDADKLSQVPLVDMYRAARIAGVPLDIQGPGITSEAADVFKISAGLKQAFTAYVKASEGYYYAKEHGTAGLMRAHYGLYLRWRRMRLKDMSLQPSFKAAQANCPQDAMDIDSANKELRAEWEDLLEIEKEGGPSVAHYAKKFGAKVARDNPKIVASVSAVLLPGVIVFSTRPEVIYGVRKAGDRVTELVRAQLQEKWEQWQQVRSDWNMGPPEAPISALYDNYMHDSRAWFKPLGDDDDVWNYKQIQELKSKQASFEREHAAWRKRAETGAPGPWQIAQAMSAGASGLGPIAMQPEPEPRSPLTAQQADLLKRYDAAMQSAKQARAAKDPNAPTDSAVLTDPKVTGGLALQTSGREFYFLWGFLRWRTVFVNGVRWDQPRVPTVQEEMEGMRMQMQRQVDMKGIGVLFQ

>YP_005627674.1

MQLSRAQQAEMMSAGLGRPQDSCCIDLRWGFFFDGTNNNFHRDQPKKAHSNVARLYDIFEADRRKPEFVRRYAAGVGTPFKDEVGDQGLGIQEKAGLAAGWGGEARICWALLKFLDNLNYYFERIDLGEALGQSDPATVRRMAQDITIPSMELRKIAGDETEMLRQISMMASLQSLTATALNLPNHRGRRAVLAERRAQLRQRVQTWQRAQPKPKLRSIRVSVFGFSRGAAEARVFCSWLKDACDGGGGELTLCGIPVQLDLLGIFDTVASVGLANSSRLWSGHGGYASEDDLQIAPYVRRCVHLVAAHEVRGSFPLDAAAGVNGEEVVYPGVHSDVGGGYEPGEQGKAFIGDSIDDSAKLSQIALCHMYREAMAAGVPLNLSASRLSKETKAAFKVDKGLIDAFNGYVAATGSIKASTTVALTQAHYALYLRWRRLRLDDTAPDGMAQQPFVTRARTYKAQDVTDLLQTNAELRQEWAALQQDEKDAAYSSEASVAHVLRSTLAPIAARDDIVALVWGEKMTQWREVKPAWNDLSPLDRRIVRLHDDYSHDSRAWFKPFGAASEEAWKRQYRQRMNRLEAQDNAWQQWNRDVQPVIDDAVRKAQKHPGSFQPTPEVRPMPPLVAGQDLKDLKQWRSNGGVIPTEQDGRESYGMFGFLRWRTIFVPEKSALAHSIDAVDETLEQIKQLPGKAKQAVGDAVESAVDSAVEAGKDFVGDQVRKLIPSGLPRM

>YP_004594328.1

MSDTAESTLNALKALIAAFQTGATPVAVKTSTAEAASLPPKFPGPDDEKEKTPADNSTQQKAEYNERGRLPASRTQTEGNYARQYLEGYVGESDHQSDKKTEPGCPASLHISLFFDGTCCTKEGADEIYGSRNPPLTNIGRLYHAANWKEQDETAENDGYFSYYFPGCGARFPEIGEEHYSLDGELFANGGEDRINQALLKTYSSISYAVNKTAIKDSELTRYRNSMATVWPFSRLTQKFDRKSALDKFCDDYLGCVVNQWPRQPTRLHIQRSQRRIAKIKLFVYGYCRGAATARAFARGLESLLDDAKMPLDATNMLPAGTVVPGGPTLQGIPISIEFMGLLDTVSAVGVPHILPSATGHLGWAANSLRLPTTKGFLKSCYHFVAGHEQHGDLPLDSIRGPDGKYPAGVVEVVYPGVHADVGGDCKPSELGKVQTDARSLLSKIVLHDMYAAAFDAGAPLSVPRDVLPGGAKNKTYRVMQGDVADQFKISAQAIALFNAWQQAGAKTETPAVNEAEVVAIAVAQGRDPVAALEAARKKADGDKAKAAATAAKSPENGPLGKLPDPVYVPLKAKALEVMLADQLSWITAWRTERFASPQYVDKYYQRKLFYQQALNESSGALPSATAPVESEVPREVPFIDKDRLDRAAWGYDFAHTGIVALLGQVLLDVIPTTQTDQLGGPLVAAQESEYIQIKKSGDARRAALMGSPTMLDFYDNYVHDTLAKYNHDPLHSSFSHGYFASRTIYDNDDDTWDSVKQFGQRVKAIIDINVGDILVQARTDMKIQLKIFIAANSI

>NP_899682.1

MQDWSDSSFETQMQAAQKRQELHPLLAGCPTCEQKPWISVFFDGTGNNGEIDADKKKWSNIYRLFQGHADDQTRGIFPIYIPGPGTPLSVSNAGWLSKLRDSGALGGGFGLGMDARMDKAFKLFSRNLADCQRVSRIDIAIFGFSRGATLARAWINLLLKECIWEKGKPHWRMLNAKNGVSAEICIRYVGLFDTVESVGMVAKNWSPSQCMTLPNVVERCVHYVSAHELRGAFPLTTVADTAGAPPGEERVWPGMHSDVGGGYRPNEQGRFDTLSRLPLNAMRLDAYLAGVPFLSPAELKGNMVENKHVFDYFEYDAELKNLYDHYKSQISDASSDLDRTIISHMKLYYGWMKLRQDGDIDSLYGKVREERADLAKELENELSDQRKYLRKIPFENLSRKENVEWARLKISDFRKYREKLESARETYGKNFRPYELNSRQLTYWDAWEKSVVPDEKITKFFDFYVHDSRAGFTFNSGDYLEPREVLERKVCSVEPARRGKQIAASNIGVDHQ

>ZP_17126626.1

MSNIIYLKIVGERQGVISEGCGSESSVGNRYQAGHEDEIFVFSLQALVSSAVAGVNHQGIRFCKPIDKSSPLFTQDINNNERCTLDFTFYRINRWGRWEKYYQIEVRGASVTAWWMQIRLDGIAEELITINYDYICSKHLIANTEYNALLTPENDNQLFPATLPAVKKPAPPIKKREITLTIGVFFDGTGNNLLNTNLRMQKCNPESYGLDARALTEFSQRCMKKEGFDGIEVGSYLNYYTNIRWLYDLYHVERIPEAINDDVQRKFYIEGIGTENNKADSLLGLGLGNNDTGVIAKTDKAIALICQLLNNLINEIDVKNSTLKHLQFDVFGFSRGAAAARHFTNRVFERDPALVNGIRQVFANSAYSGKPAGEVRFLGIFDTVTAVGGVMDGFDPHDSNNLQVKLALPPGVAKHVFHLTAKHECRYNFCLNSVKEQWPEMSLPGAHADIGGGYNPLEEEYLFLTRPAMQTVSSDIPVQSTDVYRRTVREAERLHTHPVLAPVLPSGILKIESDIDECIPSDQYHNRKKCVAAAATFRRTVSNDWSKVALRVMYEVAKEAGIIFAEIDSKNKELAFNPELNTLSERVILFAKKSLLSGHQENMLFDRDELKIIGKYIHCSANWNAVNYNIKSPVISEVAIFDSFSFVNRPDDNWIRTIYNMSGEKLK

>NP_231061.1

MDSFNYCVQCNPEENWLELEFRSENDEPIDGLLVTITNQSAPSNTYTQTTSSGKVLFGKIAAGEWRASVSQASLLTEVEKYASRKEGQESPVKKRAAAELDAADKDTKQYRFTTIGDFWDEAPKDEFLQKQHKGIDVNASAEKAGFRLSHNQTYVFEIKALRSYMPVIIDTDEFNLVNSYTFALLSKLAYATNDFNRDDGKTIDNQGAISTVISQLKRKERPTYSGDLQAKWLLEEIPYSKALSAQYYAEDDVGSEGYIIFNDELAIIGVRGTEPYFQSKKPPVDNTKFKIIKAASGMAAVIADKIESATDSPGMKDLIITDLDAAQIAPEEFGGTYVHRGFYQYTMALLSLMEKDLGLHKIKKFYCCGHSLGGAGALLISALIKDSYHPPVLRLYTYGMPRVGTRSFVERYQNILHYRHVNNHDLVPQIPTVWMNTDVSEGFHVLDVFKSRVDLMRKMLTDDDDDNYQHHGHLSQLLTYNSNNQVLLTPKQTQVTMLDLANLATNDSVAMVDGLSDASIVEHGMEQYIPNLFEQLTALSDESLMVHYQRAISALEQEIATLQQSYLTVKQAWIESIGNGTPTMNIGRLMSEMHSINKLIENRNKIRGELRQIVSDPQRMPATKFLISQQTLPDEIKVQIR

>YP_345882.1

MTSFEKDLQSPLGSRMLTCPAGGKWTSFQLIDEFCSGEPYAGLAYIVTDSEGHKYTGRLDEAGKGKVNNHFAGPVTLLFDQAYEGKEKLYSYLQGRPHYPLKITELQVRAEATHYLNPNATRTRERPEIADGGDYFQVEVRHLVRHASHLPPEVYRDYPLDSGCAAIMREHGKLGVALMPQRHTILEVRPLRALRPILSTAPAFCALNLYQLALMATLSYCPFGQKPKVPCAETRSVKFLLQPSVGNWFGDALPKGEELWKVDSAQTKAYYPLYEDVPYSARLEIIPFDPNLYAVNRHTPERDPEHPASVHFLDDIGSKDSTDTQAFITHNDELILIAVRGTAEIVADGLRDADALQVPFAEGEGQVHRGFYEAAKKAAAFAVNYLEKFYTGQTLLICGHSLGGAITLLLAEMLRRRPEGYKIQLYTYGAPRAGDADFVKGAADLVHHRMVNHNDPVPSVPGSWMNTKADIYGTGAALTFVNVPLGLSVFVAGITNWTGEAYDHHGRLRHAMPVEFGRQQVSSILWEPGCDTITQHAACDVAIRQRHGLPDRPTLLKQIFDAGHHSMTGAYIPACWAVLRRWQEAQESNRTLVTEREFALVESALQRITDQLRRQRSNLPGRPDSYVRSRKNIVEALNHEIENIRTTRERLASLRHRRLTTTDVYGSLAEQPERLAESLPRWRLHPENLATEQLAMAPEAAEDDPLLVTLYGHRIGAPHTFDIDSII

>YP_610877.1

MGHNQFLKEVNHPLNNIMLVCPVRGVSTSFQLVDEQGHGAPYAGAFFEVVDMDGTTYKGALDTEGRGEVLNHCRGPVSLRFASEYAGGDDGYMKLQTRDFYPLKITDIQVRAEQTHFQNRDGRRTQSNPAAVSADEFYQVEVSELVCHICHLPPRSLSDFPSDLGIRRIMGKHCEWGVGLMFGKHTVLEVRPLRALRPVLSMDQEFCALNLYQLALMATLSYTPFGQEPPGHPVKAKSVNFPYVPTVGNWFGDALAKGQEIWRVDTKQQTEYFPFYEDVPYSQRWEIVPFDPELYEENDPALGEDQKNPARIHFLDDREYSDTTDTQAFMTHNADVMIIAIRGTSEKIPDLLRDVDALQVPFEEGHGKVHRGFYLAAKRALQFVEVYMDKFYQSQQLIICGHSLGGAVALLLAQMLRTGGYSGPLQLYTYGAPRVGDSTFLASAADLRHHRIVNNDDMVPNLPLPWMNTRYEVIATGAVLASINFPLGAMVMRTGLVNQDGEPYGHHGELQHFMPIQLSNQESSAILWRPGCTTITAQPACNYYLEKVDGLPQHRSVSLADHFMVSSYIPACWAMLRRHQQALANRTPAVTVRELEAVDTALASISQQLRERRSRLSRGDYYPRSREPSLTAIEKELERLQATRERLASLRRAPVSEADVYGSLAGQPQLAEALDRWQAHAASTQAAPLAMAPKEQDINVVTFDELFASLDDPLDLI

>YP_001349297.1

MRALSPLLSTGEAFCYLNLYQLALMSTLSYSPFGQEPDTQPVETDSVSFPAQPSVGNWFGDALARSDELWQVDATQAGGKAYYPLYEEVAYSRRLEVVPFDPELYPEVNSPELGADQEHPARLHYLDDAKKRGGTDTQAYVTHNDELMLLVVRGTASMADVLRDVDAAQTPFEETSGKVHNGFYESAKVAIKFFATYLDKFYSGQKLVITGHSLGGAVALLVAEMLRQQPEKYDIVLYTYGSPRVGDKTFVENARPLVHHRMVNQNDPVPSVPAAWMKTSWRMSGAGVLLMLFNPAIGGAVVLLSPVNIVGEPYTHHGKLRHFMPVSFADGHKSAILWTPGCESITERGGAAVCAKALAARNGLPERGGLLRQMLDNADHKMVASYIPHCWASLRRAQEALEEGRTVVTPTEYERVGEALQDFKAQLREKEAAAYGSAREQQIMGPLRAEIGRLQQTAVRLKALSVERVSETKVYGRVADRPEALAVSLERWLAHSFNRSHEQLAMAPPDADSNERAIAALVGGHVPGAVFDLDIDASG

>NP_793265.1

MTVQSIKTWEKPFFNNRIHACPMSGHSVSFQLVDEFGDGKPYAGLAYEVIDYEGVLYSGKLDANGSGKVDNHYCGPVVLKTCEEYVGEDDFYTALSGRGSYPLPITELQVRAEKTRFSNKSGVRTRSNPAQSEADAFCQVEVSELVKHACHLPPIVDRNFPPNEYVRNLMQTPPEDVGAGESGFGPKPARVYGIGLLPNKHHVLEVRPLRALRPALSTDSEFCALNLYQLALMATLSYSDFGQKPDLFKVKSDSDEAYVVEADTVSFPLQPSVGNWFGNTLSKFEELWQVDAAQAGGKNYYPLYEEVAYSKRLEIVPFDPDLYPEVNRPSLGDDQEHPAKIHFFDDTTSKNGTDTQAFITHHDEIVLLSVRGTASTSDAFRDLDAAQVPFEEGVGKVHNGFYGSAKAVINFVTSYLDRFHVGQKVIVTGHSLGGAVAFLVAEMLRRRKGYDYDIVLYTYGAPRAVDETFATAATALIHHRTVNHTDPVPSVPTTWMNTSKPVYITGAIVTFVNVPIGLALFGGGIANLTGEPYTHHGKLRHFMPVSFADGHKSSIIWEPGCDTITEHAACTVALQQKSGLPKRGGTLRQIIDNANHKMVVSYIPACWAVLRRYQESQEFKRSLVTEREFRWVDDALESISLQLQAKERQFTMQALESQVEAQKQALRDERSKIQETRDRLGTLRFTKATETQVYGLAAAVPEALAVNLERWRLHAINTTLEQLAMAPPDADSHDRAVASITGGHIIGAPSHFDVDSFA

>ZP_11083947.1

MTAATPRTLEGCVDCKMLKYEAEIKLVDELGQPLPNLPYVLWVGHGPKKIIRQGKQSSGDGVIVEKELPPGPLYLMLEADALADVLQEPHRHLRLSRSDYGTPVQREAEQQGRLPRYARFGQLVDRLPALFEEEQQKAKDKRHPLPPYHFPHGDPAKASAARRPLYIFTKAGARSIKITLEITPLRAWVLMLEHSPEYNLGNAHNLALMAHLAYAGGDVNEANTKRVKEKRKTDPKYIPSASERAHSITHFFVEQMQDLSRLPYGINALSKAALVKDVPYRERYETPIFIDCTELGEQGEAPPSDMIGGSYDTQFFYVQRPEELIVSWRGTASLSDGLTDGNFTPVPCQGMNIADQGKAHEGFYNQFAAVNKHPKPSIAKVYTDIANSLTGKKLFICGHSLGGALALLHAASLKAKNPLLYSYGMPRTFTESALRELTFPHFRHRNERDKVTSLPPGRGVDTPLARMPVIGKVTSAAALLVPDKDPYAHHGKLVHFDYTDTSYRLRQERGQDVMAPLPVQTKLLVVPHLAAEGKVNMPKEAKEALAKAYPSGDNPSHGGGAHASSHGSGEYAGYLQRRFLGLLDGQQNLPDPYVPKLAEYVSTLKDYKAKIAPEVNWREHGAAQIDNQLQSLLPSGVTESEQFAITRLWQAPRK

>ZP_11985746.1

MTTAITSLKHKYPHAPAEEQLPYWVEILLVDEQGDAVSDMPWKVESHHPGDGIIKQFTYTGRSGSDGLIRIDMPHGLELKLTLDADSLAKEMEKRPLRVGRDAEMDSLIRQLAEDKGYIWHYAVIGELCKVTPSIKLESGEGWPPYHFPHGKSFKGFIIRTNELERRHVIEICPFRAWELVLHHQKDYSIANGINLGVAATLAYADDNALSKSSITNFFINQCQDLSRLPRLYKDKSSWNTLVRDVPYSERYSPPVFMDTSIDSSSKEDKDNQQGTISTKNDVSNVKADGDTQLYYVYNSDKIIIAWRGTESLFDAGTDIAFNPVKTESCDVNKTQCAALVPAGKVHNGFWSGYLRLGRVFNNELDELLTLIKSRDLFVCGHSLGGALALIHSAALKLEKPLLYTYGMPRTFTRNAIMELSDITHFRHINDNDPIPAVPMEANLDNEWYKLWGLLGGTLGFFWSLGELMAYQLVAWGDCFWHHGNTVAFLTVTQSREWKEYKRNLPSPAGGITIRKRLPVKAKLYLVPVLAEQEMQEAGQKQKEFQASLTKTDLTRFFPQGGNPERGVNINIFEHFMTSYMPYMYNKLLELIDKAGIVEKRTFTEHLYNIDLFKMQMEENKGCIPDKEFSRNKIFLDIENMLDVSLSSTLSMPSGNDTLLRFAKYSEEVMENA

>AAL18491.1

MSKLSPLDCLDCKNMLKHWIEFQLVDEQGKPLVNMPYRLKIRGNPLLGRKGVTDGNGLLREEDMPPHPVTLYIGAQPLADEMEQRPLREIRGEGASVVKPKAEAEGYQYRYVTIGQISDGLPALDDWNDPKKIPPPYHFPDPEPKGYQVHPVNRRYVLEVCPFRAWILLLHHQKEYSIVNAYNQCLMSVLAYAGGDVDVEGSVLHFFNRQMVDVSKLPYKVEALSATPVVYDVPFSKRYTRVEFIDSQAGNNKQGDTKLFYAASKKDMIISWRGTVSLDNYLTDATFQPLALSCADEKALCSEFIHHGKVHKGFWEAFSLVGKLTVPSEETKVTTVFSDISDLVKNKLLFICGHSLGGALALLHSAQLKEHNPCLYSYGMPRTLTRSAVEELSSIIHYRHVNEDDVIPAVPFEQDMDNVFFNYWAPAGYDWAVMKLLSPSPIIQAIKQATASKEIYLHHGKVVHFFQANSCPEWLISARNASLITGVAERILDNTTKLYLIPELNQETEKDFSLAGERQNALFNQLSQQEKDKLFVENRSADLKGGFGFSNHSSYKYAGYIDKRLRELCEPDKITVYQDSQRQFKAKMDSYKMLIPDNVYYRNLYFLDMDKQLIKSLTVSQQEEQGELALQHYCDKQELSV

>YP_002151060.1

MTFRYDKYNDPYCPDCAKHNAWIEILLVDELNNPISDMPYTLTVSGGEKRTGKTDRNGIVRETDLPPTGGRFSINAQMLADEMEKRPLRVRRNGSSKIRYDATQRNDNYRYLTIGDLCDATPKILKWDQPELPKYHFKKQQPNGYMVIFRNERWVFEVCPFRAWSFLLHHQKDYSITNACNLSILSVLSYASFKANNDKEPNAGNYLGSIEDVFFNQLFDLSQIPQQFAKESFTPIIYDVPFSERYTDVEFIDSAKNLDTQMFYIANNKEVIVVWRGTAGKTDIFTDIKFKPVKLRQDMGIEGYVHSGFYNSFRTMDGKYKLRPKIGSKNDDENPLNLIKGLASNRKLFIAGHSLGGALALLHAIKLREYNPVLYTIGMPRVLTLSITEQLGDIIHHRHVNEDDPVPALPFEKDMNNIAFISDHDWLGYSIEIAITYIDWKHQGMASKAIDQLKTNFNIKSDTFIHHGDAVHFYKPTSKTSMYKYVLASVGYAPSLLVNMEIDKKRYLVSDTDEKLYLVSELLPDHRLKQEFFELYKLPKTASPGVTGGGDHSSRKYTNFIISKIISLVKYNGLTESAYDIRAELVNQKRLSQAKYDESSYLLDLDLMVADTLLPTLLMPEGITALKRFKYANDNN

>YP_003467415.1

MSNANSIYCHDCSGMLKNKIEIQLVDEHNKPITNMPYTLKNHKMAREGITDGNGMIHEEHLTSSPLRLFLDGQKLADEMEQRPLRLKRYHEYTRSTESMAKISAVFAESQKSGRQYRYAKIGELVDKIPVIEGWKEEDPLPSYHFPDSEPMGIEVMPTAFERYRYVIEVCPFRAWSLVLHHQKDYSLVNAYNLSLMSILTYSNDNSGHWGSVTHFFNKQLLDLSRSPYQVNDERFMPVVYDVPFSERYTKVVYIDSKVQGNTGHTQLFYAANKQEIIVGWRGTEMTETQDLMTDGTFQPIELGSTANGVSSGFSEKGKVHKGFWDAFHLITEIKVSEGNDKKTVFEEIIKLTESKKLFVCGHSLGGALALLHSAQLKSYNPCLYTYGMPRLFTQSAVQELTEIIHYRHVNENDFVPSVPFNKDMDNVAFQAGRKFLGYAIEVFDAAGSLEAKIIKSNNSDNDPFLHHGKLVYFYALPDSESKFYLLPELNEETIKTTQKFIKKQKNVDRYIMQDIHSFFQGNENPTGKRGTGLFDHSSTLYAEYIDKRLRELCSLSPDKKLQRKPEDLSFLESQPNSIRAIFQRAKTEEYFFLREIDNQLAITLEVTQKDERGPRALKRYFEKR

>ZP_17666205.1

MADTSQMPRIAPLSYNARGNPVHTWTLTPSHITDPVHCILPPDGVLPVIFVPGIMGSNLKSKPEKGKGKNKAAPVWRLDAGFMGKNMWLALNWINKKAGIRQQLLHPARVEVDNKGAVPERSVGTVIVPPEPDRKKTILALTKRYEERGWGEVSETSYHAFLLWLEDTLNSEFMPHKWPQFDIRPEHLHTRSVEPGPVQVTELKPEMPIAMPGLGANLAAQLPSIISDELAARGGYRMPVHACGYNWLDSNEEAASRLAVRIDELMQQYGRNCQQVILVTHSMGGLVARRCGQLPGMADKIAGVVHGVMPAIGAPVAYRRCKVGMRDEDPIAGAVIGPTGQEVTAVFAQAPGALQLLPTQDYAPGWLRLIDERGAPAMPRQPVKDPYEEIYLRRDRWWGLLREEWLAPKDGDPITWETFAINIKKASSFHQRIAGSYHPQTYVYYGNDDKYPSFETITWEMRRGSRLNGPYASSPDAFTVSSLQMPEVRDDGRSPLYVGGQAESVMPPRGDPNAPVKTVQTSYWELHCRMQDGAGDGTVPVSSGRAPIRQIRQGSVRQQVQAPGFDHEASYANPLTQQFTLYSLIKIAAKAKRPLCAG

>ZP_18859374.1

MVNKSIAQSGSATSPTTNKAGTYVEVPLQDTIPIIFVPGIMGSNIFNTALNKPVWKLGNGGGMVGTIYSQMQKSPATLQNELDPLNTRVDTSGDIKVDSRLKLTEKTLRERYWGTVHWDSYGGILTYLQMVLNNVDLNEKPIYGGGVGGGYAVIHQMKQQEAVYEWKSLLNQGESKKWTPQEPFISINQQEIEHLKKFHFPVYAMGYNWLQSSENGAATVAQKLDKIKQEYGTRFHKFIIVTHSMGGLLTRRLVQLRGGDIAGVVHGVMPAEGAAAAYRRLVAGSSEGGLVSAVAANVIGKNTEHVTAVLANAPGGLELLPSKAYNNGRPWLFLNGSGLMNGKDLQTNVVSLPKRDPYEEIYKADGVWWEMVKEELVDPANMVKKSNPNKSVKNIYKLKINEVKNFHDKITNKYHPCTYVNYGHDPKHSSFGTLTWTLDRPLRGLTAEQMKKLPRATAKEIGTYRQKIVQEQMKKIKEGKGADANNRDLALENNGIRYISLVSGNLGVFSISNQNAPGDGTVPYQSGCAPLKQAGVKQVFKMTGFDHQGSYNNTQVKRSVLYSIVKIIKENNIQPKYR

>YP_840525.1

MSDNTNQSGGNSDFSDDTEQVVRQVGRTDKDGASVGHFTLTPASDTRQKELLCDVRPIIPVIFLPGVMGSPLVNKDTGEDIFFPPNTDGMLGKAGALPALLGMWFRGASTRETLYDPTVAQVTPFGPIRAGKLQKDDKEDQYVDEAEARRRGWGSVYRSSYQPMLAWLEEQLNEPKYMGKHKGAWIETDPDGTEWTLKPVLGTEPADYGATDAGAQKRGQAITEECAEFEHFAKYRYRVYAIGYNWLQSNEKSAKDVIEGLDVEDKKTGKKMRLMGIKEIIAENHSGKAIILTHSMGGLVARMAIAMHGAAGLMHGVFHNVLPATGAPIAAKRFRTGGGSEGGINGFINGALLGSDADEFVAVAANAPGPLELLPMPDYHNGDPWWIFARLDGTPVMKLPQQGDVYNEVYINQKWYGLVPEQSTSLLDPAGIVQKRLDNQPVKMSLSENFAKTMLFVVRNQEKIKDTYHDKTYVAYGDGALTPKASTASSEGNAKPKMEKSEKLDDLLAWGTVIWKGNIPPDVTEEELRAARFLGEKHDDSHTGTLRVHLDSRNVTIEFEVQKVAKLPPGSDAPDPQKNGIVPGDGTVPVWSAEAPAHSAEGGAARGIQMVFDQGGYVHQDSYKHPWARWALLYSIVQIAKDAPAC

>ZP_09628467.1

MATDRIHLPIGEEIGGYAGATVALTPSTDKRRISLPVPPDWVIPIIFIPGVMGTHLRMGKKRQADLDREDNRAWRPDEKLDSLSRRNDPPKRRQLNFDPDETEVDRYQITEDAGKFDMTGEATANSDRRHGNVPDGLPNIGLLMSAPLPPAAEQWKAKRGKQEATAAQKARWRGWSEVMFESYGTVIKLLEARMNELLTPTGDVSLGWKMPLSIPVLGVNPREWGCAGDPLTEEELRRVGNCWYPVYAMGYNWLQSNGVSAGKLATRIDEVIKMYRANGRRCEEVIVVTHSMGGLVARAMLNPKYGKGIGEKILGIYHSVQPPIGAGAAYKRVRTGIDDAKGSVPAAIARAVIGKTGKEVTAVFANASGPLELLPTASYPRGWLRVQTSEYRQVMALPIASDAPLKAYFDELDLHKKLGTAKPAPPVAVGDPVYDIYAREPRAWWRLLNPDWVNPADKKYEGADPNKITLERIAETQKFHKNIKDLYHPTTYASYGEDPSQKSYGTVTYRVNATDLSRFGDPLSWTFESEDGEGRIVVRAMNRQTLQLRLEPPIDAGDQTVPSEASASHVRATMVFRQTGYEHQNSYNDDKVLASTLYSIVKIANTAPWWNK

>YP_003739804.1

MAEEKTAGAGDGQPSTCILPEWEHNGLVKWKGIQNQKKSLDAQVVLLQPPIRVIPVIFLPGVMGTNLMSDGSSKREQPIWRGDSEVGVYFKWAGQDGNRRRKLLSPDTTKVDNRGDINQNIYSPFSDDGNLFPTRRERGWGGVLSFSYGKFLSVFQGALLDDWQRDMVNYAARVGGKGGILSQLVGARLSEDKATKIADESVMTQQELDHFRNFLFPLHVYGYNWLQDNKTSAEGLVDYIEEVIDLYTHQHCHGMAFPAGQEKVIIVTHSMGGLVARYASQISGAQDKILGIVHGVIPDIGSPAAYRRMKVGAKQEGMAGAVLGNTAQELMPVLARAPAPLQLLPSAKYLGGAPWLTVEGGNEDGTDIKLPKRRDPFSEIYLNETLWCRLYEADIIDKDAAEIKRNWNAYSDLISKAVQRFIEKLDNKYHPNTYAFYGHKIGSDGTLSWNRITQVYLKKTGDNDLKLPNNYREVPLPPHGAQVYRLGSSNTPGDGTVPVESLNAIRQSRSIKSVLATNVDHQGAYNVNSLNDIPNKPALKFTLRAIVKMVQEVPVP

>NP_250201.1

MSSEPLEPNQDVIIPRSRDSLGRPVYKAQLTRTDNQSEKVALIRQTAPLPVIFIPGIMGTNLRNKADKSEVWRPPNGLWPMDDLFASIGALWTWAWRGPKARQELLKAEQVEVDDQGTIDVGQSGLSEEAARLRGWGKVMRSAYNPVMGLMERRLDNIVSRRELQAWWNDEALSPPGDQGEEQGKVGPIDEEELLRASRYQFDVWCAGYNWLQSNRQSALDVRDYIENTVLPFYQKECGLDPEQMRRMKVILVTHSMGGLVARALTQLHGYERVLGVVHGVQPATGSSTIYHHMRCGYEGIAQVVLGRNAGEVTAIVANSAGALELAPSAEYREGRPWLFLCDAQGQVLKDIDGKPRAYPQNQDPYEEIYKNTTWYGLVPEQNSQYLDMSDKKEGLRVGPRDNFEDLIDSIANFHGELSAAGYHSETYAHYGADDSRHSWRDLIWKGDPTPLETPGATLNDDENGTYNSWFRRGLPTIVQGPLETGNPLDASGSGGDETVPTDSGQAPALAGVKASFRHGSKGKGQANTKRGYEHQESYNDARAQWAALYGVIKITQLADWHPNDKGGT

>CBK84496.1

MDDNKNSPPNRSYHRPIWDDDGTFHYKVFSQPKDKNHISVCLKPPTKVIPVIFIPGVMGSNLKSGDKKVWQFSLSSLKKWPLAGPQKRKQLLDPTTTVVDDSGEILNDGADGKKFPSRHARGWGSAFYMNYGEALDRLQYLLSDDEILMDNYFRETQLQTARQRFIGVRIGNEPQEQVLSEEEVAHGHKFLFPLHVFGYNWLQSNADSAALLGEYIRKVLSAYHGRLAVNKVVLITHSMGGLVARHYSENMGGQDSILGIVHGVMPDLGSPAAYRRMKIGERGITGMIIGESAEKLMPVLAQSPGPLQLLPGMAYGQGWLKINSKETQLSLPVADPYEEIYLNKTAWWRLCEQDLLLDDTRVECNKYEKTISNIVKKFIEKLNGKYHPQTWLFYGASKSNPSDGFLTWEERIPLSVKEAQRSRENAANPFELSPLRSHQLISSASPGDGTVPITSVCTSSSRIQGVLATDVDHEGAYAVDPVDLSRSVYSDLSDALVFTVRSVVKIVQQVPAP

>YP_542179.1

MTSTMHMDRADKNKVGTVEIEDLRVIPVIFLPGVMGSNLMDKKGKSIWRYDDSMSLMGWSLPTSGPKERKRLLHPDRVEVDNRGRIPAPPDAQEKLIQLGQQYPEDPSDKEAMDNYTQAVRDILDNIEPEAKLFGSRKDRGWGEVANASYGSFLDVLQTALYRDKPTKKGETLSATYQQLLDVPLGLEYGPDSLDEEYLEVIRLYQFPVHVVGYNWLGSNMLSAIRLQEQIKKIVGGYQKRGMKCHKVILVTHSMGGLVARYFSECLSGNTDVYGIVHGVLPSIGAAATYTRMKRGTENPESNPEGYVISHILGRNAAEMVAVFSQSPGPMELLPMNDYGEEWLNIVDRDGSTLTLPKNLPIKEGIAPEERTYAELYLNRENWWKLVDENLLNPFNTSLNQKQIDTDWNIYENLITESVNPFHKQIAGKYHINTYSFYGRAKLGDIPEAHLTQENVLWKGSLSMGKKSDISLEPKFIDGRLDLNEVGNIRTIKDEFSPEEQAWEINTDDGDTYVKIGQRFTLRDSCENGDGTVPLRAGQIVHKNILERLAVQVSHEAAYRNPVSQAFALRSIIKIAQEVKKDGKMSYSD

>YP_005629797.1

MTTNENGAAASAPTGEDEWDAPQERVATRYVDAQGHTIYAWNLTSSKLTDPVKLYMPSHRIVPIVFVPGIMGSNLKALKDIYRSGQDGKASSRKKIATKGQRIWNVDSMTSPVRADNSISWPGQDAAARQLMLNMDAVEVDDRGSIELRSEESNVYLPDEGRTRSAQRRREEIRQARLDDKRRRGWGTVSWCSYGAFLNWLEEQLAGATYRNGKPSLAFLELFKHVGSSPTGAIHAPPPLTEEQIKKLVKFRFPVHAVGYNWLKSNLDSGQYLADKIAAIRKHYTDLGMDCQKVIVITHSMGGLVARAASQACSADKDILAVIHGVMPTDGAGAFYKRFVGGLTGEGAGVFGSLVGKVTALVLGASGRETTPVLGFGPGPMELAPNQLYNGGKPWLFIKDAHGKTLLSLPERGNPYEEIYRSNPWWQAVNPAWLNPAGLAIDALQRHRRLLDKAESYHKQLAQKFHQPTYAYWGNDAADHKAWGTVTWRAVVANVYDPKPGDVFFGDAMPTRAWSERPGLPIVGHPAQWRWSPGHDDLPANEGLPERYLLDGKGQTLRCTIQPAADAGDGTVPAGQSGAGVMRGSPAVACRTVGYDHQMSYNNDSVRAFVFDSVVRAIEPIVVKA

>ZP_18494315.1

MSNKPEPIRELECKFDDNGSPSWDSFPSHKNCQVRGGCDLPPHLPGIIILVHGVNSTGEWFSVAEKKLCEGLNKRLGLTGTSYELETNKYLFDDKIDAVPLMSRDLPDVNINKSPVIRFYWGYASSKGNEDRYIIPLANKKGVDYHQLKRENMPYANIMAQGPFFWGGGPFQNGTNNLHSLWSEKGFKERVGGVKVQWFNEDKDRLLTNAPPRKYYAHAAKRLADMVDSIRNKYPKDTVTIISHSQGTMIAMAAVAIAKNAPDALFLLNSPYALDHNDLNGASLPADECISPEGRQNTLSAIIDKVACRKNHLLSLGYEGLCVGQTADKKNWRPDVALVGENGDGLAERDNHGRTYIYFCPHDRVMGSRPLRSIGWQGLPNDSQGQPHPLLKKHQGYLFQRMLARSTPCGETPNPVTPFAKLPDGKPFWDDKGDKYQSSSFTYPDPPEWQTVFINAEMVPEPIEAAKLADFDESRVGAEHDDREIDGWGEINPDKKSKNDNTYDNYINLYPDQDIVTGFKNTGTESEPRMVPVTRKETFEEKDLRLRTYVSQPTDHSTLPMRADFMSKVVAYDLPIGYCDATWDKEFMADLRRKADWTQGEDPYLFTGIPDNVPEPDIISRETVTDKFNKEKYKLPMYRSVNKA

>NP_755270.1

MSTNKSEPTRKVDVHLTDNGTPFAYSMTSHKNVKVRAEVQPPLQLPGLIIFVHGVNSEGEWYDYAERSLCAGLNQRLGLEGEHGLKENNYEGGFFVNSDKSEGGWEHTYEIEGSQKKWVSGPRKITKGGDGRSPVIRFYWGYRAADNETDTYAIPLKNKKGDNYYDLPPESRKAKGPWFWGGGPFQNGCNQLVSLWSKTGFNNNPSLLGVPLPFSTQVLNGERDRLLSDAPPRHYYAHAAGRLAKLIKTIRNQHPEDTVTVLSHSQGTMIALAAAAIEAPDALFVMNSPYALENEPTTYISYPIKEIISRKARSATFADIVKKVAENKTRLKQQGCDNLLAGMSSDGNSWIPEGKTHNGLPERDNHGTTWIYCNPHDRVMGSSPLRSIGWQGLPDTKDGTPHTLFKQAGDTLYVRILGRNTPCGGTPTAQTHFSNLGDGKPFWDSTTTLLQRATWPDPDSGQTLTINAPQVPEPLTAEELKNFDQDYARDEKQSGGAGYAYGQINPETKKPVDTDYRYYISLYGYFDRKMVPKKDSGYYQSGPGSKEDRVKYEKQSQEEMLEEVRTYVQRPTDHSTLPSDERFMSRVVAYDLPIGYCWHSWDKAGLEELRRQADWLESDDYYFSGKLTVPPIPPAIKQDVAEDAEQRKAEEKARLRNV

>YP_006577256.1

MADEKKSPYCPRVVSENDIPVPKSLSLTAQTCRVGIPRPMPGIVILVHGVNDVGEAYQNQERGIIAGLNKRLNRSDMYAHEWHDFIMMHNEEAQKKIKAPGRSPVIPFYWGYKPVTHDEYRADQQRYRNEVSKLKAEAHLPFDAYQEDDAKKKAELGNDGQGAFKYQNDNFGNALDVNYAKGGGTFANATTNIPDMLGPGAGGVALAAAGFMTLHANGGDFTHPIFPNPHRIYQFFAAQRLADLILTIRREPVTENDVINIVAHSQGTLITMLANMLVKQAGYEPVNCVILNHSPYSLESRLAEDIQPGHHQTSDARVQTFKNFCALMATHYKGGEITEADILAMEAACALRKPSDNPLRKDERYRRDNNGRVYNYFCPNDGTVSLKNIQGFGWRGIPEDIASHIPNLYQRVFYQHGEVGAKPDGKTFSLPSARTGDADYSSIGNASYTAHDVIVNGEELPVKFTFMLQGAGNHKDDDPKTSDKPYTAYIDPDSPDAYISYSAKAYAIKRTQSATYVVSRYQSLSWRPGHVLTPDELKMESYERGVEVIKGVVTGTKDFPTVTLTWLRPREALEKEWAKSDPVSYSQHSSIVMSEYAPSHAMAFDLAIGQCRSFDFKAGKFWEELLHRADWRDPLNKNQQAVEYYRSGILPDETTKWFMNRPDDILPTGNFGVVNEFNNAITVKPSKDLAAGNQEIANLQWDMPKTKSDRELGLQNQVSALDLRPYGAGQGSL

>NP_248951.1

MNDRVRSRTIVSAQSITLPKGGDVHLVPPPPKPCVTIVVHGVNDLAGCYERIERGLCQGLNERLDMPPTLPGGQANPGYLTPAGYSLPADDEGKAENPDVVYYRRKFASGAGGAAVRSVVVPFYWGFREEEQYINKTAAHGEWLDRNGNRLDKSGTKEGGQFVNATTNLPDMWGQGFNGKLFGFISLDWFGGTMTHPLFSAAGRKYMVLAAMRLAMLIKIIRKRYPDDTINVVGHSQGTLLTLLAHAFLKDDGVAPADGVIMLNSPYGLFEPLNEKLQGWSSQQTREARLATLKGILEFICGRRHPVPALSSVALRNCQGYGAIGGPGWVGGQGCQTTIDGERLSFDERDNRGSVYLYFTPQDQTVGLANVQGIGWRGIAEQVKGLPGRTGLPQGFHQRIFTVRKRNGEKEKIGGHAPPHVYPLLLAGEKTWEDTGLGGKDRFGRANFDQGDSVLLTAPRLPLPTEARFDFDGAVTAPGENSASGVYQVRDTLDPIDAAIGVSNGGWKEKDSGHAVAQQVDAALAYRYGRDARSVERALNEGKELAQQTHVFSARELGTGMVLVTRAETPYEARLRLQTAEGHLEPLSFHSAIPNNPEHNRRVLAYDLAIGAGDSVDDVVFYQYLCRVADWRLDWKASDKGIFSQGDASVDLPDEEVRALYRAEESKNSQLIDATVAYRKSGEFPVVVGNRLPSLVGTQTILDRYHEQAVRFGGTI

>NP_794788.1

MNGADEYAVAQGNTRLIPNLNTTCKMEVPADLPGVVIFLHGVNDPGASYESVETGLCQGVNERLDRPDLVPGRYGGKYKEAGNVPYEKRDSDQKVILDDPDTYLYRRDASDPKTHSLLIPFYWGHRAAPDQIKRDDAGDPFRMRNQFQDINGNRLDRHFAKAGGFIANATNNIPDVYGEGFRPNLKSIALETFKPDNALYFGHSPARHYCVLAAHRLAMLIREIRRVSPDETITIMGHSQGTIVTLLAQALLVDGGDRCADTFIMVDTPYCVLPGNTPKDQDTFSTLVGIVTAITNMPHTQPAMSELRDAKTYCGRSGSRWLPTQGIRKNKVGSMTVFPERDNRGKVYLYFCPDDTTVSLDDVQGIGTYGMPDALPDGRMAMMVLPQLRFYQRMWTKRHRYGEAILIGNTPQPELMRATGEARYPGSSFGAGMIARAPILEGQERLINAEALSPPHEPEMFGGEASRGTPTTSGLDRPDDVAKGVALGKDEATFMWVRMPSEYDSPNMSQQEAQNAFNALSDDPENHTRALRKIKSTTNSSSHHEREETPREARERMEKNRDAWSENSYHSGILRSPENHRWVTAMDIAIGQAKCLDDPAMRDVLIAIADWKIDKKVFEYIEKLPDWVRLSHKAQALVKASNDYYVKGKFPPSSLVPLTPPPLVGPALNAGTVA

>YP_443720.1

MANRPGDRPVPIPRDLPGVVIFIHGVNDPGAAYATVERGLCQGLNERLSRSDLRPAEYGREYAEAIKAKDRKSPFFDSKIANDPDMYLYRRAESGGAHSMFLPFYWGYRASDNEIAKINHPGEIKSRVADSDGNLMTRGQYQDIHGNRLDAHFGKGGGFFANATNNIPQMYSRGFEPDKLERTVMQNALAGNTIFAGKSPERRYFVLAAARLANLIKTIRTIQPSALALEHGMDPQHETITVMGHSQGTIITLLAQAMLKQQGQRCVDCIIMVDTPYSLQFTKDGSQQTGHAKLKTLVDIVNAVTSEPHTIPDLAELMIDSAHSCGRAGQNWSQTQGKRLDKSGKHWITFDERDNRGKVYLYFCPEDTVVGLDKVRGIGTFGVPDEVPADGAAASRGKTMPAMTALEPKRFFQRMWTRLERDQDGRGRRSKVAVGTPPARVPVRDPFQRLTPGPDTDGTMLGTLVESGKNMALQASFKRNDIRLINGEQLKPAYEPDLYGGEVQKGGQVPGHADVAGLMRPDDVTKNVALGNQYAKFQWKDVATTDDPGASIEPHKQAFNRGRPVDEQSHNWRIVPSRSLGSMLSAAATGGRYQTYVIQREETPDEVRKRMRTDADQLEANNYHSGVLLSSENHRWVTAMDVAIGQAVTLDDPDWRQLLLLMADWKMTPSAQKKITNCKSFARLDDHTQGFIDACAKYYQKGLFPSEKFVSLALPSLITSELKLDQKT

>YP_001915284.1

MTECSYVVAQANALLLPNRMGERLVEVPADRPGIVIFIHGVNDPGAGYPTVEKGLCQGLNERLSRIDLRAGQYGVKYAEAKKSPLKPGEQGYKEVASVKYDPDTYLYQRSEDTTSKLPTHSMFIPFYWGYRASDNEIAKDKRGNPTRLRSQYQDTAGNRLDANFAKAGGFFVNATSNLPDMYGKGFETTLKTRGVQMVSPDFTYFGNAPPRRYFVLAAERLAMLVSEIRRLAPDDTITIMGHSQGTMITLLAQAMLADRRQRCADCLILVDSPYSLLEPKGEEQTTQAKLQTLIKIVKAVTAQPYTRPALNELQVGQPGYGGRTGHGWTPSQGTRLDAEGKQIVFAERDNRGKVYLYFCPQDTTVALDQVQGIGTYGVPDTVHVAWKRKFYSTERTASLPAMDALKDMRFHQRMWTKLLRGGKPVAVGLPPQHIPLRMEDEARYPGGGVGPTTTASQTPLPQEGRYINGEALQPPHAPQMEDGEADARQYKSSTPLRGTPTRAGKDAPDDVSVDVALGNPKASLNQYRVFERFVDKNLSDPDLQELTQQFNANHPDLNDQTPGYDCENGDDMGYMLWRHATPNEVRAQMAHNPAALVDNSYHSAMLRSTENHRWVTAMDVAIGQAQTLDDPEWRKVLIAFANWRTPFKPSESQLPGQLTLLELANFEKLSPGAQMLAQQTAYYYTTGKFPDGVSKEPPDKYVISRTRAQRAESNE

>YP_934973.1

MNTSDFIAVREAVLQPDKGTATCVAQLPLPGVIILVHGVNSDGEWYEATEQGLCAGLNTRLGRRDEDLAIKGVAGGQMVPTTYAPEIDENGFLDRNRDSETFIRDTPFSPVIRFRWGYKANKDELAEYGSGIWLNEHNYWGGGPFANGCTSLGDLWGAGVDDRLFLWFTVQHLNPETGRQVYACPPRHYYAFAALRLAELVRSIRQKQADVPITIVCHSQGNMVGLAAAFYGARIGTVTDSEGKSAPAIADNYVLANAPYSLVEKGMGTDDWAQRYSVNSKGQWGRQTRNAREQTLANFFALIRSRIGSDQPKDEVQRVCGNGEAYDVATDRSLRCRNGRVTVYCNPHDRVISSLTVQGIGWRGMNAEDFETTQAHGVLFQRVWAQGNPVGASADGEYRYWDGESAFWHPPPRKARYSLKQGVEAGASIIGKVMTVVSTPLIWLIVLAVRAFDKSPRVNAMPDPEWRVPVNAPVLPEPHLPQGSRLGQPTAFDQDGDPDHDRLRPRLNDGGSEPDDPYQAYRRDATEVTEAAPQADADTEGRLKYEHRARLRMNDRRRGGDGEGANQAPGETTKRWDAWARKEIKSFLDQSLDQHATDHSTIMTCAANMEKVLAYDVAVGLSRLTDKDWRDLRVAADWQLWEGLDRSDAHRYFGEYFDTGRLGHGGGKRLPLHEHPDFNTTLHSAAIPPGVVDERRHAAESREARNGKPT

>CBG37357.1

MSSTGAHPHCQPCENLKHWIEIIVRDEHNQPFEAVSGVLIDAMKKKHPIELNASPILIENLAPGPAEIELDYDPWLKAAQDKSHPRNEETAKPVEEFSSSYSAHKSGPVVYQEITTGDLTKLPKEIVLPTNHQKGKAGTLKLFTDKTYILQVRAYKFITLRVGMFFDGTANNTYSAQWGKQQLENYYRKWKAKYDAECEINSKNGNGTKKEVPITALSNDCFTYPKKDNFILSLFKNDEGEMETVAGSASNELTNVQKLFDLYSQDKFFKEKNMFSHAEYITGIGTGNSTAIAPADESIVVGQGLGIGKYGVTAKVTTGIEALSKNMDKVATIVKDELGIKADGIEKLQLDVFGFSRGAAAARHFVNVVLDGEKGEFSTTFSKACQEAKFPLVYGFDWNESNELKANCEITFAGLFDTVASVVNIFSKNSPLGLDLNTHTDNGDVRLWIDPRRVRRAVHLTADPTIECRDNFSLNHLNSTDEEHFHEFVLPGAHSDIGGGYHSRLSFDNPDYLLPVLEKKLVKRVSRTFSERWDEEKTKQYVLNELEKYKVRDRLTGWKEEDYVIEPLDIRQEGKNDGGRVTGKLYIQRQVEGDLSRLYLRLMYGLAEFHGVPISDNNAKLWQDPERVDYNVEDYGGLFTDLNQKILELAKHGEYSALQQKLSIPELKASFMELNLFHHSSGDDIGMSPLWDERAGCYKRASYFCEEGK

>YP_443212.1

MNFRFAPAERPDIGVVTKEEKDAILRRLHDDDGMSCCKTLHIGIFFDGTRNNAERDKSGHKHSNVARLRDAFPQDRYHKSIYVAGVGTPFSSEIGDYGIGLQAVAGASAGWAGEGRINWALLQIHNAVHECAFRVGLSTALGVDDKNLVKLMSLDMNFKGIDLGGNAPQPGSTGDIKSRSSPGIGALKLIAAEQYGAELTWDKDTNWSQLKDDLDSSKWAAAVRAWDGRRRKILGDRRAQLKARVGDMLVKGKPRIQRIRLYVFGFSRGAAEARTFSNWLVDALESDFSLCGVPVSYDFLGIFDTVASVGIAQSAAATLFDGHGGWARKELMAVPHYVRRCVHMVAAHEPRGSFPLDLIDCSLEGREEIVYPGVHSDVGGGYGPAEQGRGRGDADKLSQVPLVDMYRAARIAGVPLDIQGPGITSEAADVFKISAGLKQAFTAYVKASEGYYYAKEHGTAGLMRAHYGLYLRWRRMRLKDMSLQPSFKAAQANCPQDAMDIDSANKELRAEWEDLLEIEKEGGPSVAHYAKKFGAKVARDNPKIVASVSAVLLPGVIVFSTRPEVIYGVRKAGDRVTELVRAQLQEKWEQWQQVRSDWNMGPPEAPISALYDNYMHDSRAWFKPLGDDDDVWNYKQIQELKSKQASFEREHAAWRKRAETGAPGPWQIAQAMSAGASGLGPIAMQPEPEPRSPLTAQQADLLKRYDAAMQSAKQARAAKDPNAPTDSAVLTDPKVTGGLALQTSGREFYFLWGFLRWRTVFVNGVRWDQPRVPTVQEEMEGMRMQMQRQVDMKGIGVLFQ

>PAAR1

MPSAARLGDSCAGHGCFPATPIIAGSGDVIINGKPAARKGDAVLLHACPCPNMPHGIHSRAISGGSGNVFINGKAAARVGDAIGCGGSVAAGSGNVIIGDSPYQSPAKSCAEQTAKKRLPLLALSPMLVPWQTLLEWAAPVALPETEKTLTEAQRKLRYQARKQLSERAAKVPGLADAGKRLGFNNDSILRAEAAQYVYGVDEFNRGARTDLPAAPVGLELIDTKKIPGLEKAVFTSKESGFGAALFKSSINNETMLTYRGTNNPVTGKMDWMTNIAQGLVGAETKQYNQAMDLARQVKRTVASPSSPLVVVGHSLGGGLASAGVGTTGLPGYTFNAAGLHTNTVSRKGGLPLDKIGDLMTTQAVDGEVLTMGQGVGKLAVPTLFAGIGSAIGGSGGAAVGGALGSLLLAGGALPPAAGKMLPLPSQGGNPVARHGMDQVIAGIEAQKSEDIGTITSKLGGVK

>PAAR2

MPSAARLGDSCAGHGCFPATPIIAGSGDVIINGKPAARKGDAVLLHACPCPNMPHGIHSRAISGGSGNVFINGKAAARVGDAIGCGGSVAAGSGNVIIGDSPYQSPAKSCAEQTAKKRLPLLALSPMLVPWQALLEWAAPVALPEAEKTLTEAQRKLRYQARKQLSERAAKVPGLADAGKRLGFNNDSILRAEAAQYVYAVDEFNRGARTDLPAAPVGLELIDTKKIPGLEKAEFTNKTTGFGAALFKSSINNETMLTYRGTNNAVTGRLDWMTNAAQGIGSETKQYNQAMYLARQVKRTVASPSSPLVAVGHSLGGGLASAGVGATGLPGYTFNAAGLHANTVSRKGGLPLDKIGELITTQAVDGEVLTMVQGVGKLAVPTLFAGIGGAVGGTSGAAIGGALGGMLLAGGALPSAAGKMLPLPSQGGNPVARHGMDQVIAGIEAQKSEDIGTITSKLGGVK

**Reference**

1. Zhou, Y., et al., Taxonomy, virulence genes and antimicrobial resistance of Aeromonas isolated from extra-intestinal and intestinal infections. BMC Infectious Diseases, 2019. **19**(1).

2. Edwards, R.A., L.H. Keller, and D.M. Schifferli, Improved allelic exchange vectors and their use to analyze 987P fimbria gene expression. Gene, 1998. **207**(2): p. 149-157.

3. Khan, S.R., et al., Broad-Host-Range Expression Vectors with Tightly Regulated Promoters and Their Use To Examine the Influence of TraR and TraM Expression on Ti Plasmid Quorum Sensing. Applied and Environmental Microbiology, 2008. **74**(16): p. 5053-5062.
