# Supplementary figures and images for: Mechanistic insights into the T6SS of multi‐drug‐resistant Aeromonas hydrophila and its role in competition and pathogenesis
Source: mLife. 2025 Jul 22;4(4):363–77. doi: 10.1002/mlf2.70018 (PMC12396204; doi:10.1002/mlf2.70018)

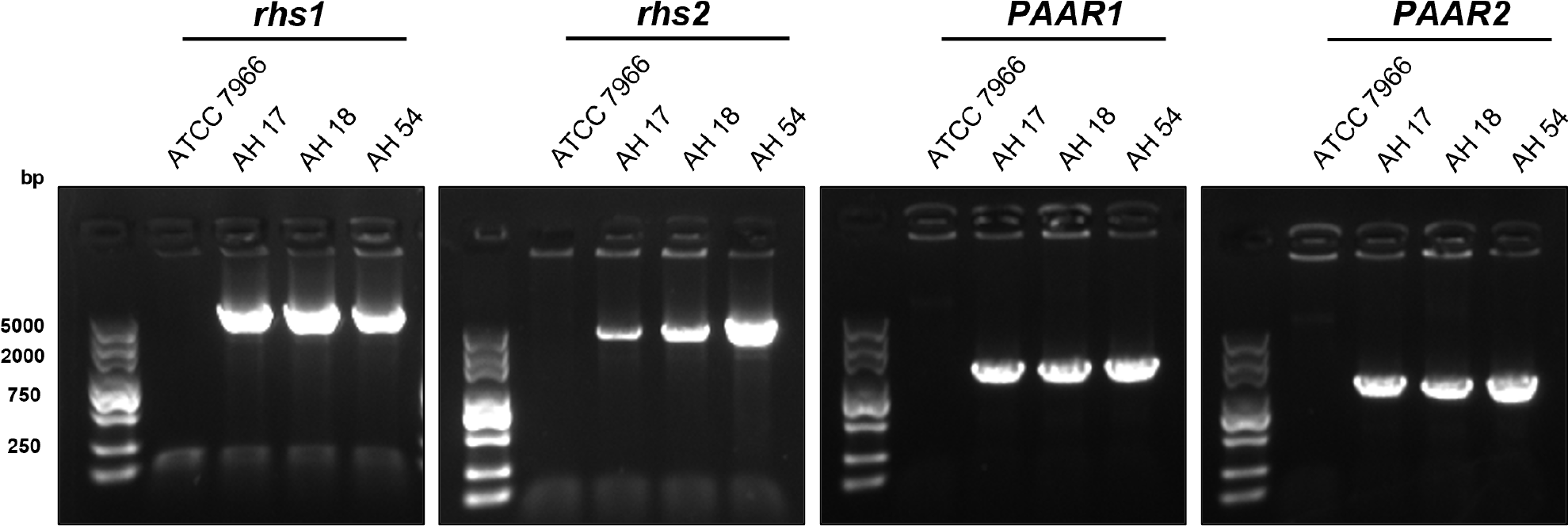

Supplement: Supplementary file 7 — Supplementary Figure 1. [file MLF2-4-363-s006.tif]

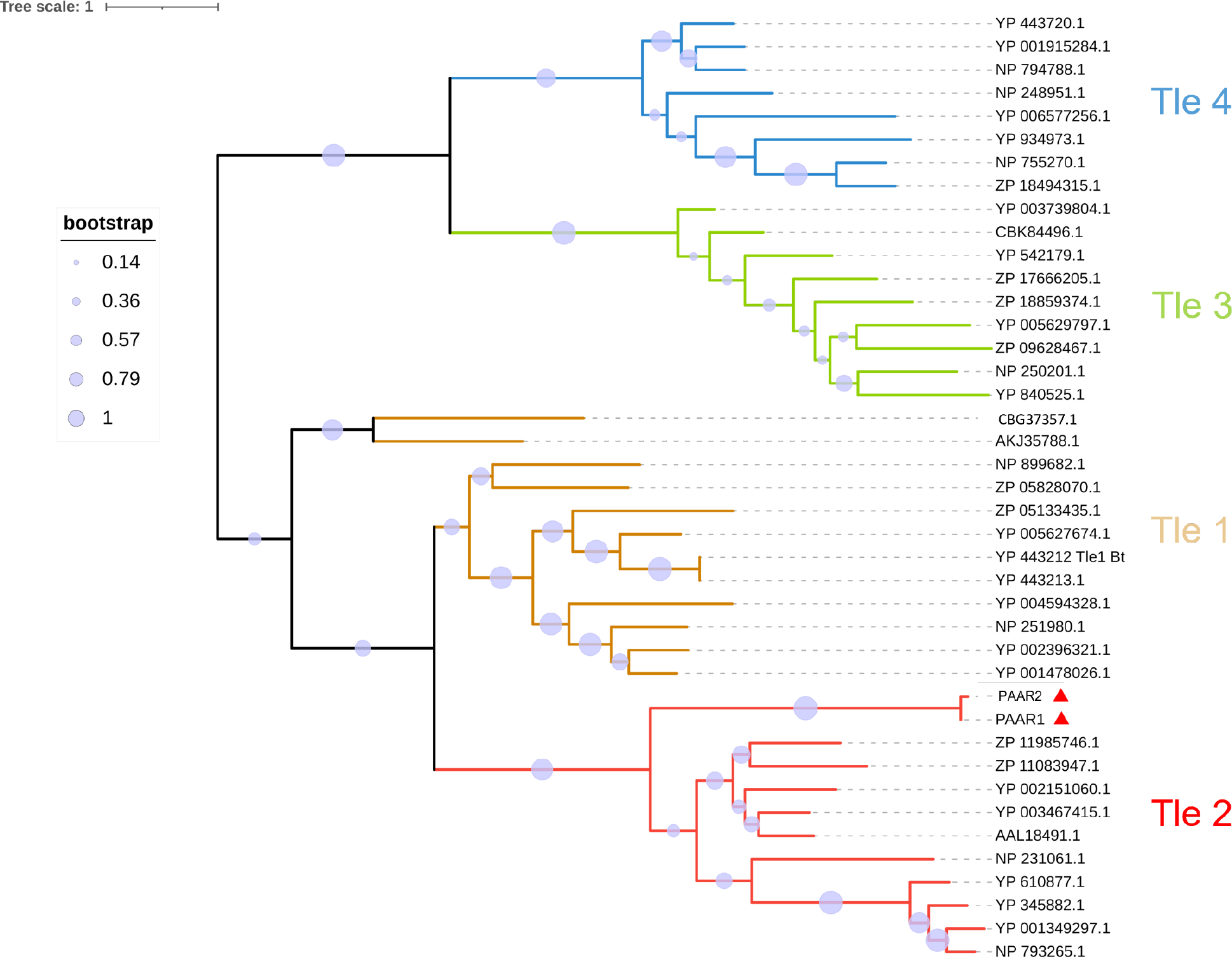

Supplement: Supplementary file 8 — Supplementary Figure 2. [file MLF2-4-363-s010.tif]

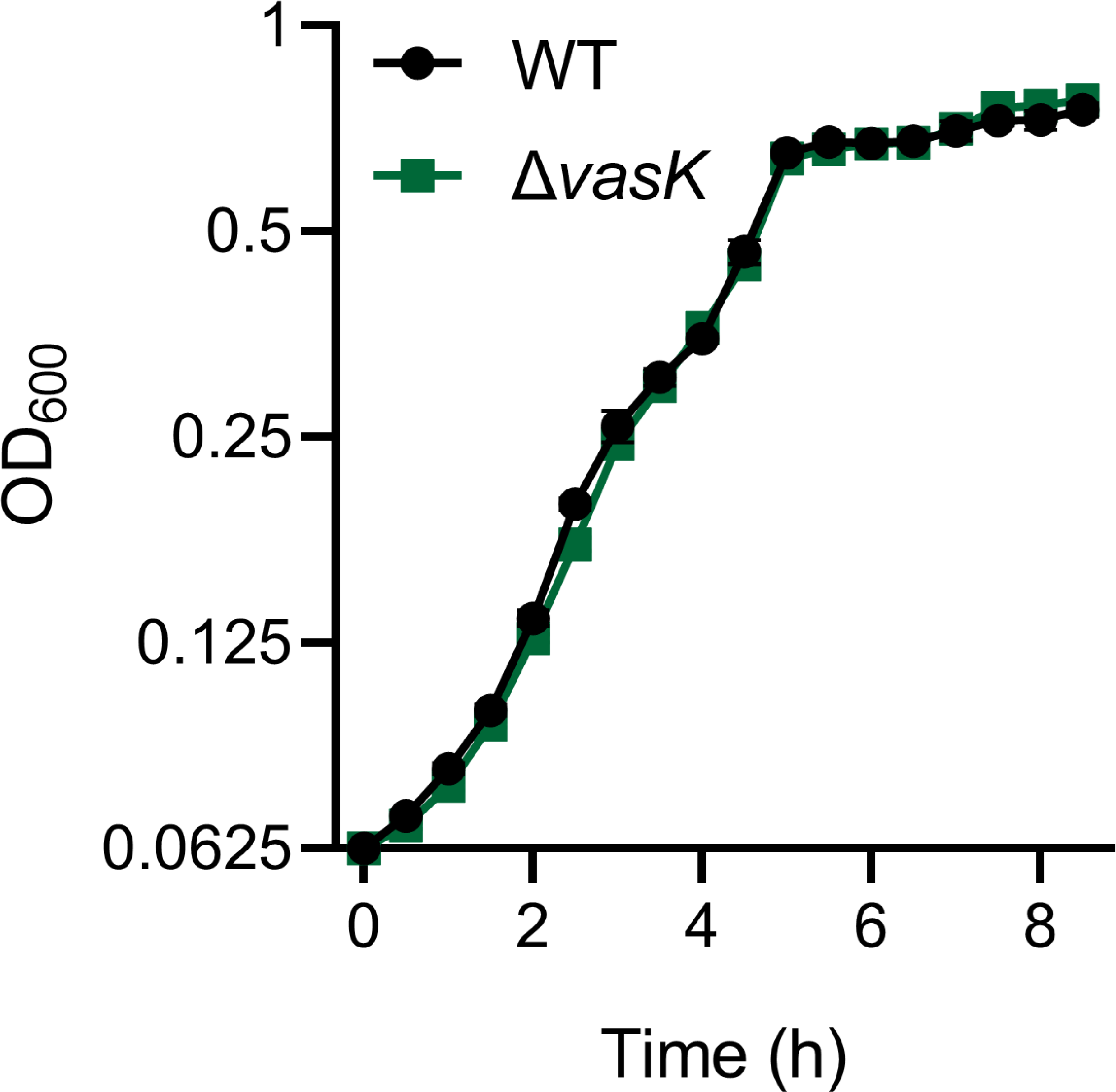

Supplement: Supplementary file 9 — Supplementary Figure 3. [file MLF2-4-363-s001.tif]

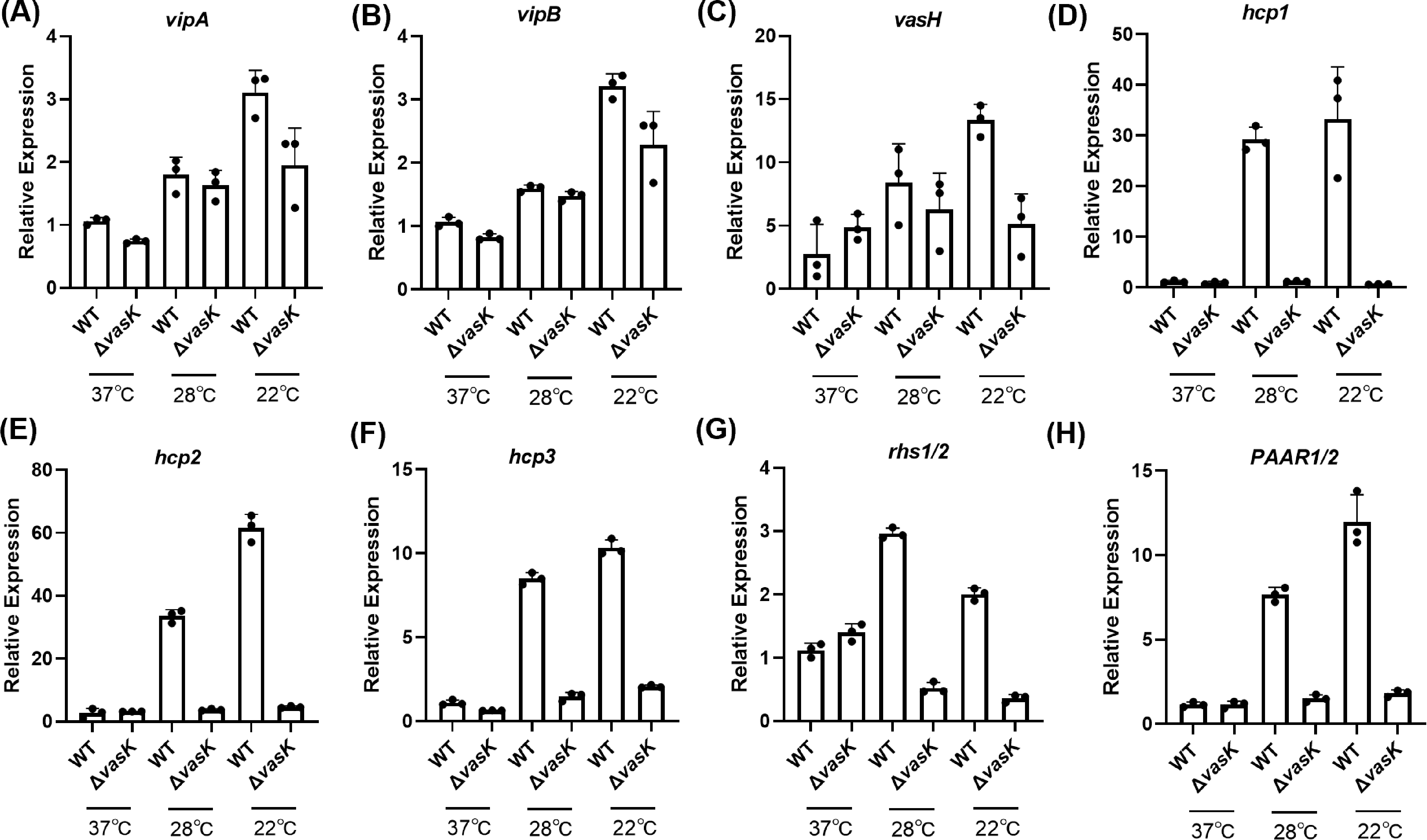

Supplement: Supplementary file 10 — Supplementary Figure 4. [file MLF2-4-363-s011.tif]

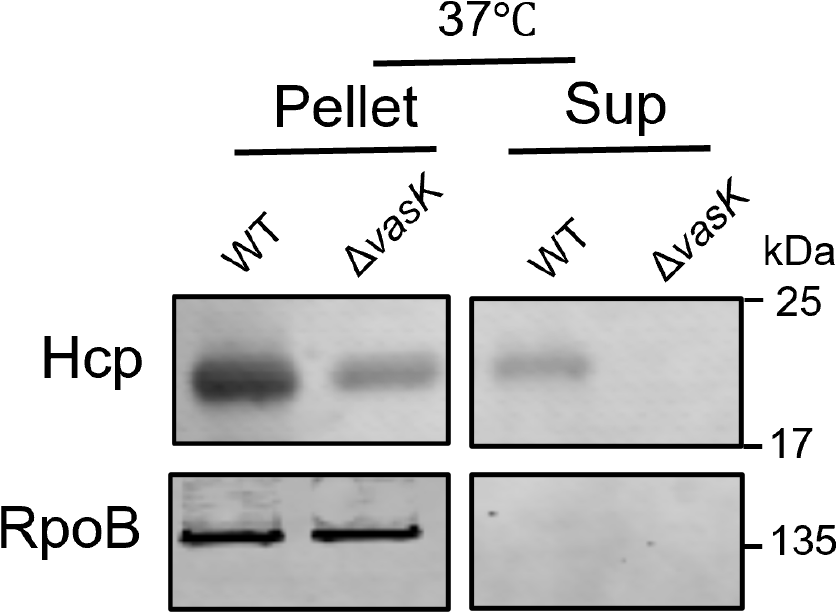

Supplement: Supplementary file 11 — Supplementary Figure 5. [file MLF2-4-363-s005.tif]

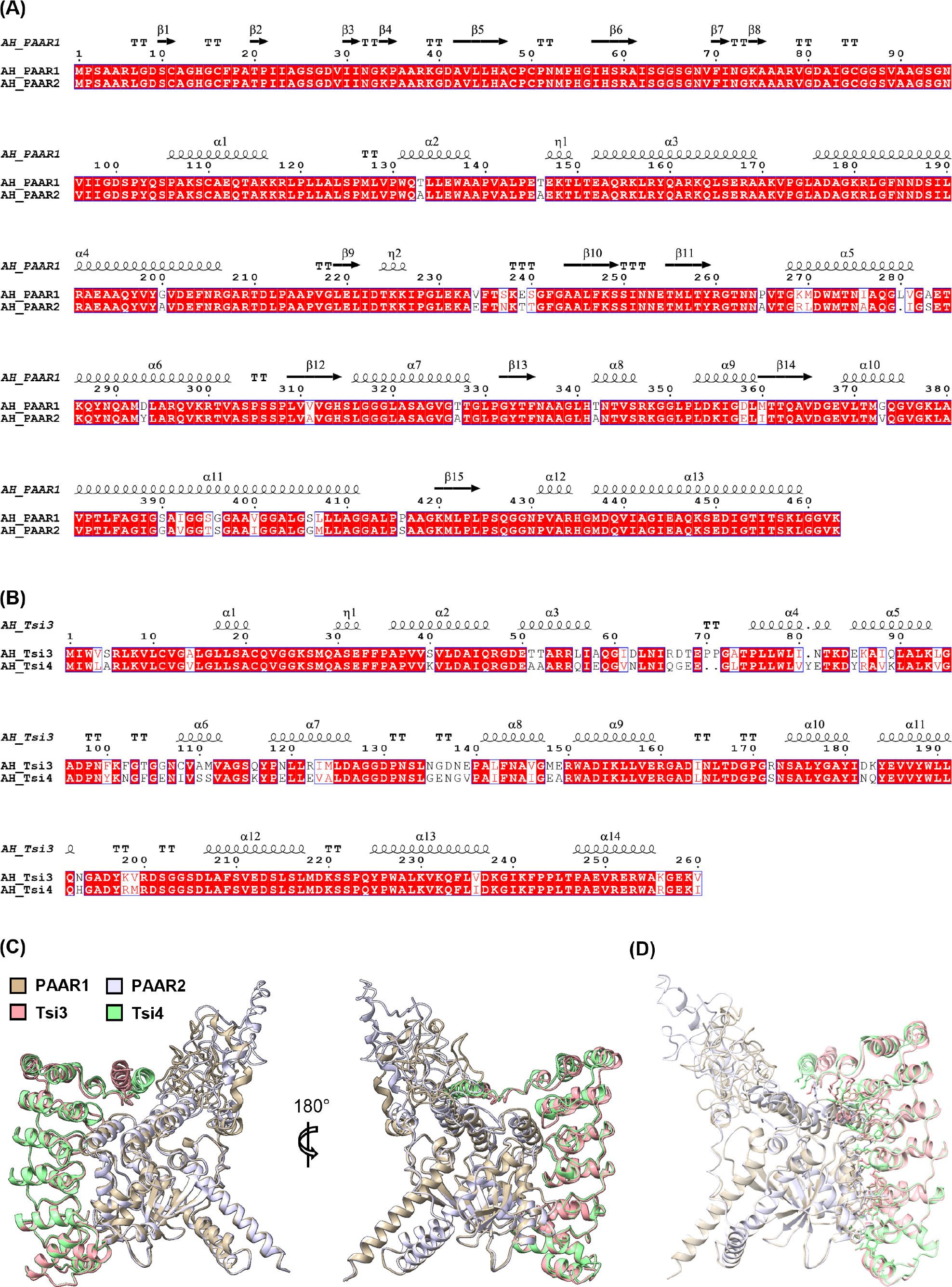

Supplement: Supplementary file 12 — Supplementary Figure 6. [file MLF2-4-363-s012.tif]

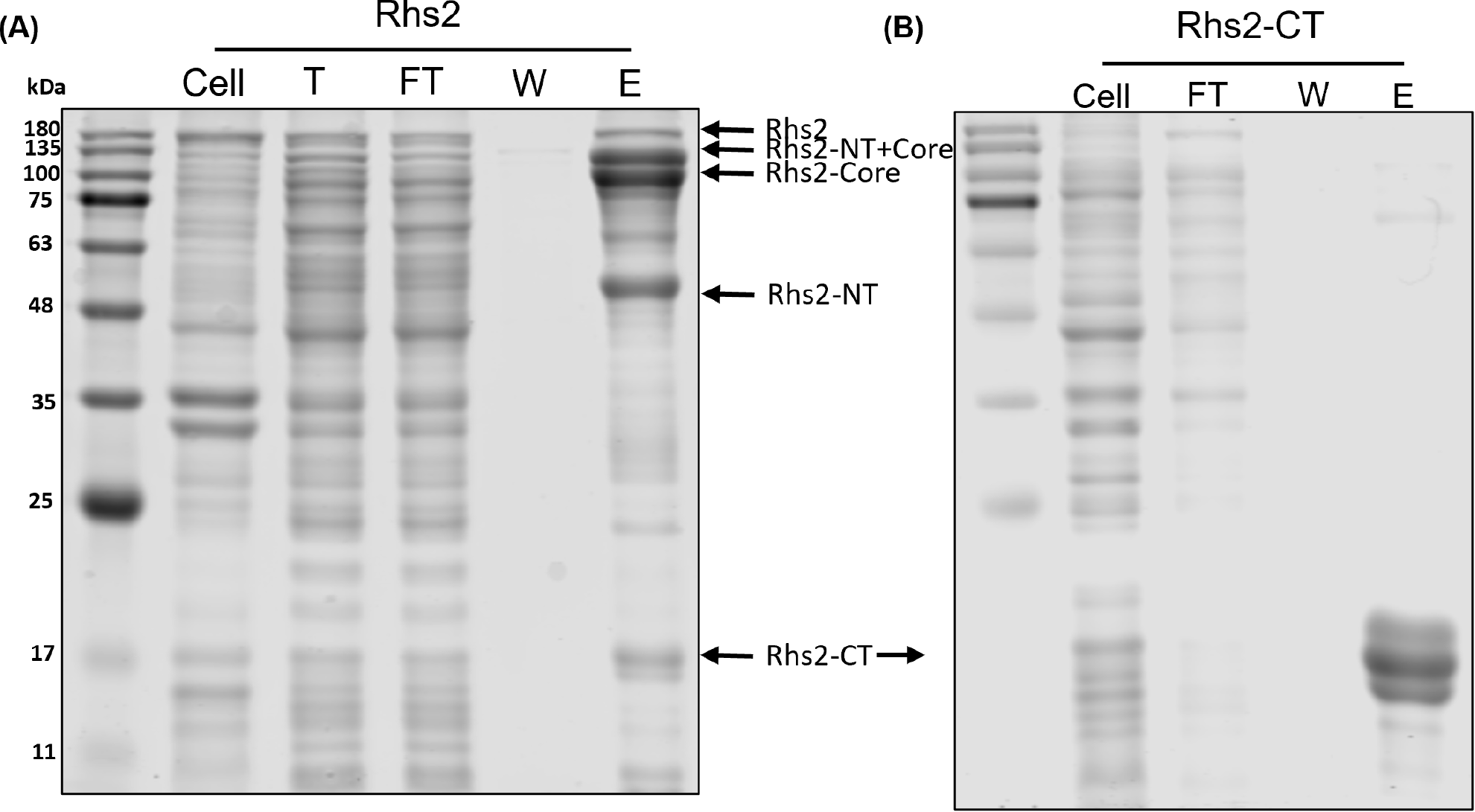

Supplement: Supplementary file 13 — Supplementary Figure 7. [file MLF2-4-363-s009.tif]
